# Maize Canopy Apparent Photosynthesis and ^13^C-Photosynthate Reallocation in Response to Different Density and N Rate Combinations

**DOI:** 10.3389/fpls.2019.01113

**Published:** 2019-09-19

**Authors:** Shanshan Wei, Xiangyu Wang, Guanghao Li, Dong Jiang, Shuting Dong

**Affiliations:** ^1^College of Agriculture/Key Laboratory of Crop Physiology, Ecology and Management, Ministry of Agriculture/Hi-Tech Key Laboratory of Information Agriculture of Jiangsu Province, Nanjing Agricultural University, Nanjing, China; ^2^State Key Laboratory of Crop Biology, College of Agriculture, Shandong Agricultural University, Tai’an, China; ^3^College of Life Science, Nanjing Agricultural University, Nanjing, China

**Keywords:** canopy apparent photosynthesis, ^13^C-photosynthate reallocation, leaf area index, density and N rate combination, grain yield, maize

## Abstract

Appropriate planting density and nitrogen (N) supply are critical factors optimizing yield in crop cultivation. To advance the knowledge of maize plants under different density and N rate combinations, responses of canopy apparent photosynthesis (CAP), and assimilate redistribution characters (by ^13^CO_2_ stable isotope tracing) were investigated. In this study, two maize varieties DH618 and DH605 were grown at various planting densities (6.75, 8.25, 9.75, and 11.25 pl m^−2^) and N application rates (0, 180, 270, 360, and 540 kg ha^−1^) during 2013–2015. Maize grain yield (GY) was maximized at a density of 9.75 pl m^−2^ with 180–360 kg ha^−1^ N during the three study years. Maize GY, biomass, CAP, leaf area index (LAI), and ^13^C-photosynthate reallocation all responded more intensively to density than N rate, but the N response differed between varieties. We established links among CAP, LAI and biomass, and GY and kernel number per unit area (KNA). CAP depended on high LAI and enzyme activities for photosynthesis, yet both N deficiency and N excess had inhibitory effects. Besides, relations between ^13^C-photosynthate reallocation and yield components were executed. High density increased the ^13^C-photosynthate distribution in vegetative organs but reduced the allocation in ear, while N supply moderated the response. Based on our results, maize plants with greater CAP, more ^13^C-photosynthate distribution to ears, and less ^13^C-photosynthate distribution to stems under different density and N rate combinations could improve KNA and achieve a greater GY consequently.

## Introduction

As a nationally important cereal crop, maize (*Zea mays* L.) plays a significant role in expanding overall grain production capacity in China (Yu et al., 2014). Progressive increases in planting density are crucial contributors to maize yield improvements in China and elsewhere in the world (Smith et al., 2004; Antonietta et al., 2014; Testa et al., 2016; Jia et al., 2018). On the other hand, increased nitrogen (N) fertilizer rate has also become one of the major crop management practices that contributed to maize yield improvement (Egli, 2008; Rossini et al., 2011; Mastrodomenico et al., 2018). Therefore, the agronomic management integrates density and N supply are of great importance for stability and further enhancement of maize yield (Wei et al., 2017). Given the popularity of this research topic, however, unique contributions of physiological parameters are still required to enrich our knowledge considering this complex field design.

Maize grain yield (GY) is determined by the kernel number per unit area (KNA) and the kernel weight (KW). KNA depends on the accumulation of ear biomass around flowering and the biomass using efficiency for kernel setting (Borras and Vitantonio-Mazzini, 2018), while KW relies on the grain filling rate and filling duration (Wei et al., 2019). Consequently, great biomass accumulation around and post silking is of key importance to maize yield improvement (Muchow, 1988; Borras et al., 2004; Ding et al., 2005; Lee and Tollenaar, 2007). To fulfill this demand, a canopy with high photosynthetic productivity is therefore required, and the requirements that should be met are a) a great leaf area index (LAI) to capture enough photosynthetically active radiation (Boomsma et al., 2009), b) a high photosynthetic rate to produce more assimilates (Ding et al., 2005; Liu et al., 2014), and c) a high assimilate transport and redistribution efficiency to increase kernel number and KW (Andrade, 1995).

LAI and leaf net photosynthetic rates (Pn) are directly associated with plant dry matter production (Zhao et al., 2015). Previous studies have reported that LAI and Pn both varied greatly with the increasing planting density (Ren et al., 2017; Xu et al., 2017; Jia et al., 2018). Similarly, the N supply rate also altered the maximum value of LAI and Pn (Vos et al., 2005; Shi et al., 2016). The relationship between final yield and Pn during flowering of individual maize plant has also been established through a large variation in stand density (Edmeades and Daynard, 1979). On the other hand, canopy apparent photosynthesis (CAP) has been proven to be more closely related with dry matter accumulation (Zhao et al., 1997), as it can accurately describe the photosynthetic capacity per unit area of land by combining factors such as leaf morphology and population (Borrás et al., 2003; Yang et al., 2010; Liu et al., 2015). Therefore, the critical CAP under different plant densities and N rates is also worthy to be clarified, as the canopy structure varied a lot under those conditions (Wei et al., 2017). The photosynthetic potential of plants was also dependent upon the physiological function in the interior of green leaves. As the target enzymes for improving the photosynthesis rate and yield, the activity of ribulose-1,5-bisphosphate carboxylase (RuBPCase) and phosphoenolpyruvate carboxylase (PEPCase) also deserved to be clarified during investigating the variations of photosynthesis (Murchie and Horton, 2003; Vos et al., 2005; Ye et al., 2009).

The distribution and reallocation of photosynthates are also important processes determining GY (Borras et al., 2004; Ning et al., 2013). In maize, planting density has important effects on dry matter partitioning between vegetative and reproductive organs (Rossini et al., 2012). In addition, contrasting N supply rates has been demonstrated to alter the biomass partitioning to reproductive organs (Paponov and Engels, 2005). Liu et al. (2015) proved that an accelerated utilization of assimilates from stem to grain appeared to be critical in determining plant productivity by using an isotope (^13^CO_2_) labeling technique. As a natural tracer, the stable isotope ^13^C is safe and easy to control as it has no radioactivity. Based on this, numerous studies have quantified carbon redistribution characteristics using ^13^C under various environmental conditions, including moisture, nutrition status, light, and soil texture (Ginkel et al., 1997; Lu et al., 2002; Liu et al., 2015; Gao et al., 2017). Hence, it is feasible to assess variations in ^13^C-photosynthate redistribution under different plant densities and N supply combinations by ^13^CO_2_ stable isotope tracing to advance our knowledge on this topic.

Studies on the interaction effects of density and N rate on maize crops have been widely performed *via* detailed physiological analyses (Muchow, 1988, Muchow, 1994; Rossini et al., 2011), mainly focusing on response of GY to its components (KNA and KW) or nutrient accumulation and allocation dynamics (Boomsma et al., 2009; Ciampitti and Vyn, 2011; Ciampitti et al., 2013a, Ciampitti et al., 2013b; Wei et al., 2019). To the best of our knowledge, however, there have been no reports of the joint influence of density and N supply on CAP and labeled photosynthate reallocation in maize plants. Besides, the impacts of these indicators on yield formation remain largely unclear. Therefore, this study aims to quantify how the CAP and ^13^C-photosynthate reallocation of maize plants respond to diverse density × N rate conditions and how they affect GY formation.

## Materials and Methods

### Trial Site and Conditions

Field evaluations were conducted from the 2013 to 2015 cropping seasons from mid-June to late September or early October at the Shandong Agricultural University Experimental Farm in Shandong, China (117°09′E, 36°10′N). This area has a semihumid, warm temperate continental climate with monsoons. In our research, the field was fallow for a season after maize harvest, and rotary tillage was performed before maize sowing. The soil at the site is neutral sandy loam, and the nutrient status of the top 30 cm before seeding consisted of 11.4 g kg^−1^ of organic matter (Walkley and Black method), 0.89 g kg^−1^ of total N (Kjeldahl method), 25.6 mg kg^−1^ of available phosphate (Olsen method), and 107.2 mg kg^−1^ of available potassium (Dirks–Scheffer method). The temperature, solar radiation, and rainfall during each growing season are listed in **Table 1**.

**Table 1:** Monthly average maximum temperature (Tmax, °C) and minimum temperature (Tmin, °C) and monthly total solar radiation (SR, MJ m^−2^) and rainfall (mm) during the growing season (June–October) in 2013–2015.

Month	2013				2014				2015			
	Tmax	Tmin	SR	Rainfall	Tmax	Tmin	SR	Rainfall	Tmax	Tmin	SR	Rainfall
June	30.8	21.6	245.3	13.8	30.4	21.2	211.5	34.3	29.0	19.9	179.7	62.5
July	31.4	23.8	419.3	344.8	33.4	23.0	519.4	44.3	33.3	22.7	532.4	74.2
August	33.0	23.4	557.0	35.0	31.7	21.3	464.5	28.6	31.6	20.9	474.1	120.7
September	27.5	16.5	373.5	8.0	27.4	17.8	292.4	94.8	28.6	16.6	392.9	13.0
October	26.6	11.3	92.2	0.0	–				26.6	11.2	85.2	8.3
Average/total	30.7	21.3	1595.2	401.6	30.7	20.8	1,487.8	202	29.8	18.2	1,664.3	278.7

### Experimental Design

The high-yield maize varieties Denghai 618 (DH618, 521 × DH392) and Denghai 605 (DH605, DH351 × DH382), which are widely grown in China, were used as the test materials. Both varieties are compact-type maize hybrids with 19 (DH618) and 20 (DH605) leaves in total. Maize seeds were hand-planted on June 15, 13, and 18 in 2013, 2014, and 2015, respectively, and harvested on September 28 (DH618) and October 5 (DH605), September 29, and October 5 in 2013, 2014, and 2015, respectively. In 2013, the treatment levels consisted of three plant densities, 6.75 (low density, LD), 8.25 (medium density, MD), and 9.75 (high density, HD) pl m^−2^, and four N application rates, 0 (N0), 180 (N180), 360 (N360), and 540 (N540) kg N ha^−1^. The combination of LDN360 is a common farming practice in North China (Jin et al., 2012). The treatments were the same in 2014 as in 2013, but with an extra-HD (11.25 pl m^−2^). Based on the results of 2013 and 2014, two densities (LD and HD) were selected in 2015, a medium N rate (270 kg N ha^−1^, N270) was added; nevertheless, the N540 treatments were removed. In each study year, the experimental treatments were arranged in a split-split plot design, where the variety was set as the main plot and N rate and density were set as subplot and subsubplot with three replicates per treatment. Distinct borders were made among plots to avoid N leaching from one plot to the next. Each plot was 12 m long × 3 m wide and consisted of five rows with an interrow spacing of 0.6 m. Phosphorus (P_2_O_5_) and potassium (K_2_O) fertilizer were applied before sowing, at a rate of 90 and 120 kg ha^−1^ per plot, respectively. Urea was used as the N fertilizer and was applied in the form of furrow as topdressing. One half of the N was introduced when six leaves unfolded, and the other half was introduced when 12 leaves unfolded. Besides, irrigation, weeds, diseases, and insect pests were controlled adequately during each growing season so that no factors other than density and N supply limited growth.

### Sampling and Measurements

Physiological indicators were determined for LD and HD during the three experimental years.

#### Canopy Apparent Photosynthesis

The rates of CAP were measured by a modified closed gas exchange system using an infrared gas analyzer (GXH-305, Peking, China). The aluminum-framed chamber consisted of two parts, with a 1 × 1.33 × 3 m body and a removable door. The chamber body and door were both covered with a 0.4-mm-thick Mylar plastic (light transmission ratio up to 95%) to ensure measurements under natural light condition. In addition, the height of the chamber was tall enough to contain plants for CAP measurements without affecting canopy structure (plants up to 2.64 m). Eight and 12 plants (across two rows) were mantled by the chamber for LD and HD measurement, respectively, and kerves were installed before tasseling on the ground. After the chamber was embedded into the kerve, the crack was filled by water to make a seal condition. Besides, two blast blowers were set on the ground between the two rows of maize (apart about 20 cm from the width of the kerve) and the air outlet was upturned to maintain interior airflow of the chamber. Measurements with three replicates in each treatment were taken between 10:00 AM and 14:00 AM local time at tasseling (VT) and 20 days after tasseling (20DAT) and 40 days after tasseling (40DAT). The decrease in CO_2_ concentration was linear and was usually measured within 1 min after closure of the chamber door. Soil respiration was assumed to be consistent in this study, and the CO_2_ exchange rates were presented on a soil area basis. The canopy photosynthetic rate was calculated as CAP = slope × *n*/*A*, where slope represented the decrease in CO_2_ concentration per unit time (μmol mol^−1^ s^−1^); *n* represented the mole amount of air in the chamber, calculated as *PV*/*RT*, where *P* represented pressure (kPa), *V* represented the volume of the chamber (L), *R* was the gas constant (8.314 kPa L mol^−1^ K^−1^), and *T* represented the Kelvin temperature (K) in the chamber; and *A* was the ground area (Dong et al., 1993; Liu et al., 2015). Due to the continuous rainy weather, no CAP measurements were performed at 40DAT during the 2014 growing season.

#### Leaf Area Index

Three representative plants from each plot were selected to determine the green leaf area (GLA) nondestructively, and LAI was calculated as the following equations: GLA = ∑ leaf length × maximum width × 0.75) and LAI = GLA × *n*/*S*, where *n* is the number of plants within a unit area of land and *S* is the unit area of land.

#### Enzyme Activities

RuBPCase activity was measured using the method described by Lilley and Walker (1974). Ear leaf samples (0.5 g) were homogenized using a precooled mortar and pestle with acid-washed quartz sand and 2.5 ml of extraction medium (0.1 M Tricine–HCl, pH 8.4) with 10 mM MgCl_2_, 1 mM EDTA, 7 mM β-mercaptoethanol, 5% (v/v) glycerol, and 1% (w/v) polyvinylpyrrolidone (PVP), followed by centrifugation at 10,000 *g* for 10 min below 4°C. The clear supernatant was slowly decanted and used as the source of crude Rubisco. Prior to enzyme quantification, the crude extract was activated by incubation with 0.2 M NaHCO_3_ and 0.1 M MgCl_2_ at 25°C for 10 min. The reaction mixture contained 50 mM Tricine–HCl (pH 7.8), 10 mM KCl, 1 mM EDTA, 15 mM MgCl_2_, 0.21 mM nicotinamide adenine dinucleotide (NADH), 5 mM dithiothreitol (DTT), 5 mM phosphocreatine, 5 mM ATP, 2 U creatine phosphokinase, 15 U phosphoglyceric phosphokinase, 5 U glyceraldehyde-phosphate dehydrogenase, and 10 mM NaHCO_3_. Each reaction was initiated by the addition of 0.5 μmol RuBP. RuBPCase activity was the NADH oxidation rate at an absorbance of 340 nm.

PEPCase activity was measured following the procedure of Arnozis et al. (1988). Ear leaf samples were homogenized (ground) in 0.1 M Tricine–HCl (pH 8.4) followed by centrifugation at 10,000 *g* for 10 min. All procedures were performed at temperatures below 4°C. The clear supernatant was subjected to enzyme assay. The enzyme extract was added to a reaction mixture containing 0.5 μM phosphoenolpyruvate (PEP), 3.68 μM NaHCO_3_, 0.16 μM NADH, 11.2 μM MgCl_2_, and 112 μM Tris–NaOH (pH 9.2). PEPCase activity was measured spectrophotometrically by coupling the enzyme reaction to NADH oxidation mediated by 15 U of malate dehydrogenase; the reaction was followed by monitoring absorbance at 340 nm.

#### ^13^CO_2_ Labeling of Ear Leaf

Experiments in labeling leaves with ^13^CO_2_ were performed in the 2014 and 2015 growing seasons for LD and HD with N levels of N0, N180, N360, and N270 (in 2015). Eight representative plants in each plot were selected at tasseling, and the ear leaves of each selected plant were encased with 0.1-mm-thick Mylar plastic bags, which permitted sunlight into the bags at levels up to 95% of natural intensity. Bags were sealed at the base with plasticine and subsequently injected with 60 ml of ^13^CO_2_. The ^13^CO_2_ in each bag was extracted through a KOH washer to absorb any remaining radioactive ^13^CO_2_, and then the plastic bag was removed after photosynthesis was allowed to proceed for 60 min.

Samples were collected at above-ground level from four labeled plants at 24 h after labeling and R6, and the plants were separated into ear leaves, other leaves, stem (including sheath), tassel, bracts, cob, and grain (R6). All separated samples were first heat-treated at 105°C for 30 min and then oven-dried at 80°C to a constant weight to estimate the dry matter accumulation before being milled. The isotopic abundance was analyzed using an IsoPrime100 instrument (Cheadle, UK). The percentage distribution of ^13^C-photosynthate among different plant organs (%/plant) was calculated as ^13^C*_i_* (%) = ^13^C*_i_*/^13^C_net assimilation_ × 100%, where ^13^C_net assimilation_ was calculated by summing the ^13^C in each above-ground component by maize plants (Gao et al., 2017).

#### Dry Matter Accumulation

Three sequential samples from each plot (as one repetition) were taken at tasseling stage and physiological maturity by manually cutting the above-ground parts. All samples were firstly heat-treated at 105°C for 30 min and then oven-dried at 80°C to a constant weight to estimate dry matter accumulation.

#### Yield and Yield Components

At physiological maturity, ears from three rows of the center of each plot were harvested by hand and air-dried to investigate yield, kernel number, and 1,000-KW. The GY was expressed at a moisture content of 15.5%.

### Statistical Analysis

Analysis of variance (ANOVA) was performed with SPSS ver. 18.0 (SPSS Institute, Inc.). Duncan’s multiple-range test was used to evaluate differences among treatments, and the significance level was set at the 0.05 probability level. The statistical analysis for each year was performed separately. The quadratic polynomial regression analyses were executed by SPSS 18.0, and the curve fits among CAP, ^13^C-photosynthate distribution ratio (%) in different organs, and GY and yield components were performed by SigmaPlot ver. 12.0 (Systat Software Inc.). The data were plotted using MATLAB R2015b (MathWorks) and SigmaPlot ver. 12.0.

## Results

### GY and Yield Components

**Figure 1:**
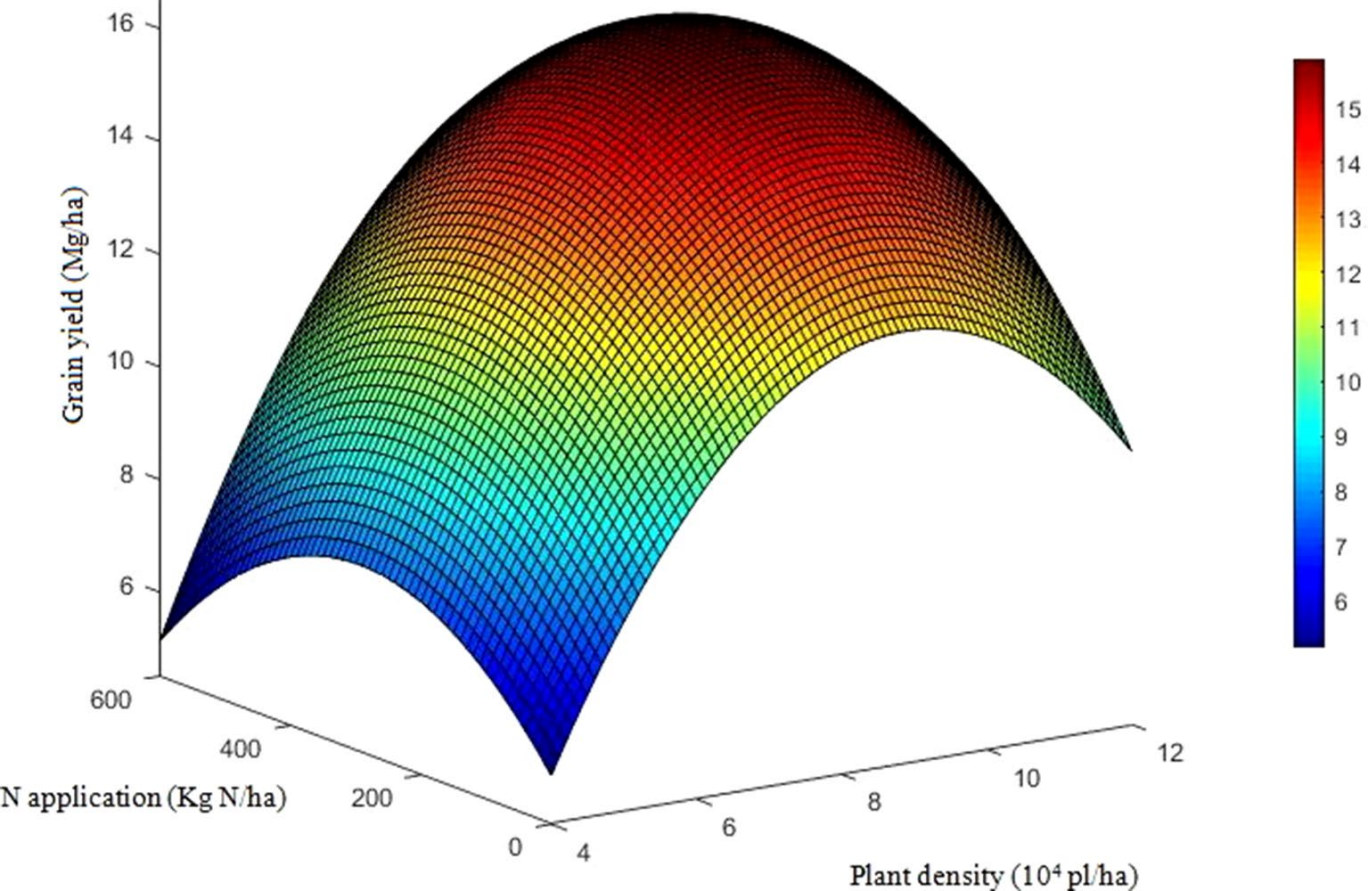
Quadratic trend surface fitting of grain yield, planting density, and nitrogen application rate. The data include the two varieties and three growing seasons (except the results for DH605 in 2014). To reflect the effects of density and nitrogen application rate on grain yield, we set density as *x*, nitrogen application rate as *y*, and grain yield as *Z*. The secondary trend surface fitting equation is *Z* = −10.97 + 5.38*x* + 1.07 × 10^−2^*y* − 0.31*x*^2^ + 9.43 × 10^−4^*xy* − 2.65 × 10^−5^*y*^2^ (*R*^2^ = 0.80, *F* = 131.1) with a confidence level of α = 0.01, *F*_0.01_(5, 162) = 3.13.

The effects of density, N application rate, variety, and their interactions on GY were all significant during three growing seasons (**Table 2**). Among the experimental years, the highest GY for DH618 (16.7 Mg ha^−1^) and DH605 (17.1 Mg ha^−1^) were both obtained under HDN360 treatment in the 2013 growing season. GY was much lower in 2014 compared to that in the 2013 and 2015 seasons, especially for variety DH605, which might be explained by the stalk rot disease during the late grain-filling stage. In general, the GY for DH605 (average 14.1 Mg ha^−1^) was higher than that of DH618 (average 13.6 Mg ha^−1^), except for the 2014 growing season. GY significantly enhanced with increased planting density until the density exceeded HD (i.e., extra-HD in 2014) for each variety. The GY was higher by 5.5%, 11.2%, and 3.5% for DH618 and 8.3%, 10.7%, and 3.2% for DH605 at MD, HD, and extra-HD, respectively, compared to LD in 2014. The effect of N on GY varied under different densities. Using DH618 as an example, the GY response to N (i.e., N180, N360, and N540 vs. N0) was superior at HD (average increase: 24.3%) versus MD (average increase: 16.3%) and LD (average increase: 15.3%) in 2013. Meanwhile, GY responded more intensively to N360 than N540 at each density in both 2013 and 2014; nevertheless, no significant differences were detected between N180 and N360 at LD. Besides, the GY response to N also varied in maize varieties: under the HD condition, GY increased markedly when the N application rate increased from 180 to 360 kg ha^−1^ for DH618, while there were no significant enhancements at N rates over 180 kg ha^−1^ for DH605 during 2014 and 2015. In order to reflect the interaction effects of density and N application rate on GY, a quadratic polynomial regression analyses were performed (**Figure 1**). We set density as *x*, nitrogen application rate as *y*, and the fitting equation is *GY* = −10.97 + 5.38*x* + 1.07 × 10^−2^*y* − 0.31*x*^2^ + 9.43 × 10^−4^*xy* − 2.65 × 10^−5^*y*^2^ (*R*^2^ = 0.80^***^).

**Table 2:** Effects of planting density and N application rate on grain yield (155 g kg^−1^ water content) during the 2013–2015 growing seasons.

Density	N rate	Grain yield (Mg ha^−1^)					
		2013		2014		2015	
		DH618	DH605	DH618	DH605	DH618	DH605
LD	N0	11.6i	13.0g	10.4k	11.4i	10.0f	12.0e
	N180	13.5f	14.3f	13.7f	13.1f	13.3d	14.2c
	N360	13.6f	13.9g	13.8f	13.2ef	13.3d	14.3c
	N270	–	–	–	–	13.3d	14.4c
	N540	13.1g	13.8g	13.1h	12.7g	–	–
MD	N0	12.7h	13.4i	11.0i	12.1h	–	–
	N180	14.9d	15.8c	14.2de	14.3b		
	N360	15.0d	15.6cd	14.5c	14.2b		
	N540	14.5e	15.3e	14.1e	14.0c		
HD	N0	13.0g	13.7h	10.8i	12.0h	10.5e	12.6d
	N180	16.0b	16.4b	15.0b	14.6a	14.8c	15.4b
	N360	16.7a	17.1a	15.8a	14.7a	16.0a	15.8a
	N270	–	–	–	–	15.4b	15.8a
	N540	15.6c	15.5d	15.1b	14.5a	–	–
extra-HD	N0	–	–	10.7j	11.3i	–	–
	N180			13.5g	13.3e		
	N360			14.3d	13.7d		
	N540			14.3d	13.7d		
ANOVA							
Density (D)		3604.2^***^		740.2^***^		1886.5***	
N rate (N)		2845.4^***^		4295.8^***^		2697.1***	
Variety (V)		947.9^***^		11.8^**^		944.6***	
D × N		144.0^***^		53.4^***^		109.3***	
D × V		53.4^***^		16.4^**^		66.1***	
N × V		23.9^***^		279.2^***^		120.6***	
D × N × V		13.4^***^		6.9^***^		18.9***	

Different lowercase letters indicate a significant difference (p < 0.05) with the same year of the same variety among treatments. The numbers in the ANOVA section represent F values. ns, not significant. *Significant at the 0.05 probability level. **Significant at the 0.01 probability level. ***Significant at the 0.001 probability level. LD, MD, HD, and extra-HD refer to low density, medium density, high density, and extra-high density, respectively. N0, N180, N270, N360, and N540 represent nitrogen rates of 0, 180, 270, 360, and 540 kg ha^−1^, respectively.

Density, N rate, and variety all had significant impacts on KNA, kernel number per plant (KNP), and 1,000-KW (TKW). The density × N interaction had dramatic effects on KNA and TKW during 2013 and 2014; however, this interaction only marginally influenced KNP (*p* > 0.05) during each experimental season (**Table S1**). The KNP and TKW both decreased with increased planting density; however, KNA increased, until a density beyond 9.75 pl m^−2^. In 2013, KNA increased by 21.6% and 21.4% for DH618 and DH605, respectively, whereas KNP decreased by 13.0% and 11.6%, when density increased from LD to HD. For TKW, it decreased only by 2.7% (DH618) and 6.2% (DH605). The responses of kernel number and TKW to N (N180, N360, and N540 vs. N0) were more intensive under HD. Taking DH618 in 2013 as an example, the kernel number increased by 10.6%, 9.4%, 8.5% and 12.9%, 15.5%, 10.6% (KNA) and 8.7%, 7.7%, 7.8% and 8.8%, 11.9%, 13.0% (KNP) for LD and HD, respectively. For TKW, increases of 5.0%, 6.8%, 3.6% and 9.0%, 10.6%, 8.5% were obtained under LD and HD, respectively.

### Dry Matter Accumulation

**Figure 2:**
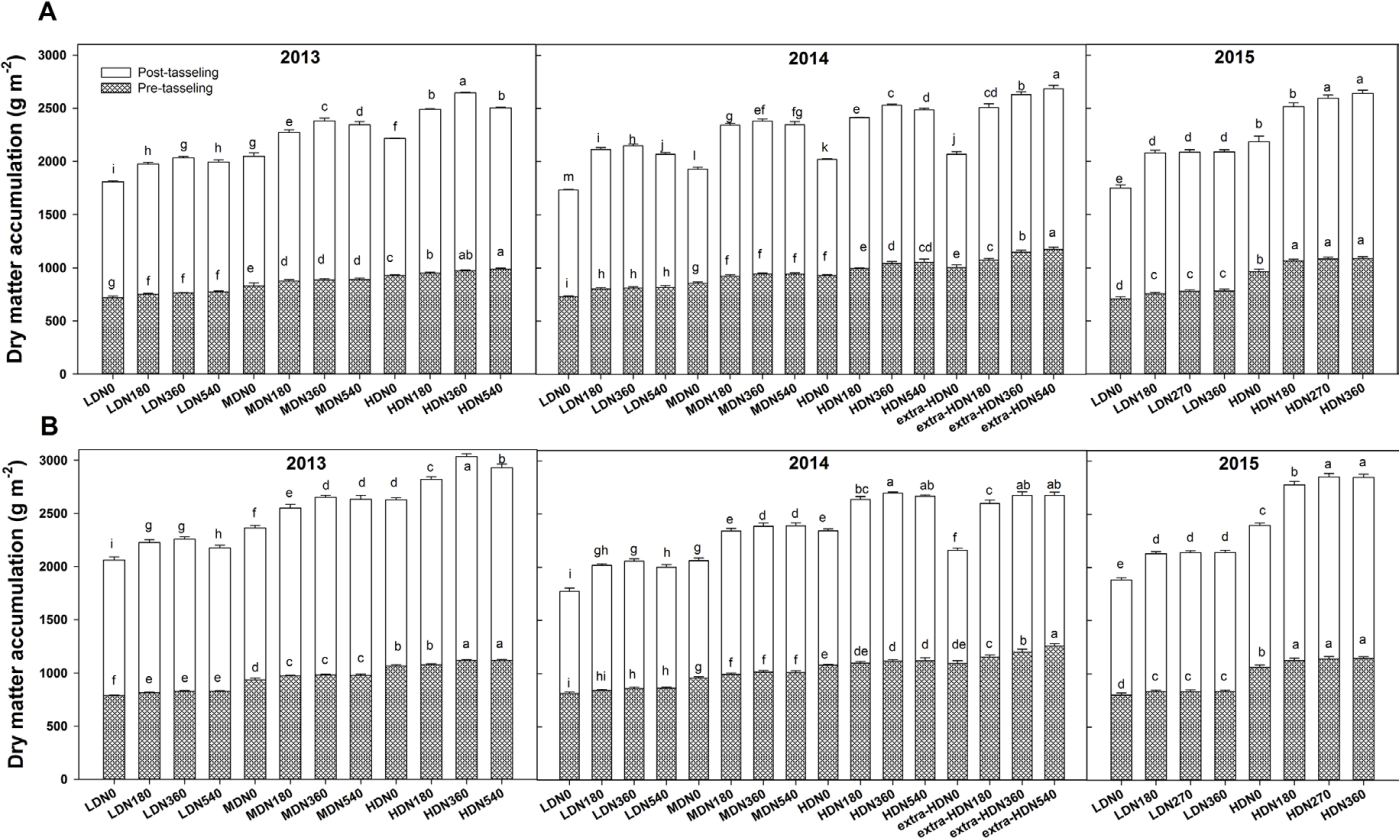
Effects of density and nitrogen application rate on the dry matter accumulation of **(A)** DH618 and **(B)** DH605 during the 2013–2015 growing seasons. Pretasseling and posttasseling represent the biomass from seeding to tasseling stage and from tasseling to harvest, respectively. The whole column represents the total biomass accumulation at harvest. Different lowercase letters at the same growth stage indicate significant differences (*p* < 0.05) among the treatments. LD and HD refer to low density and high density; N0, N180, N270, N360, and N540 represent nitrogen rates of 0, 180, 270, 360, and 540 kg ha^−1^, respectively. Data are presented as the mean ± standard error.

During each growing season, biomass per unit area at both tasseling and harvest stage were significantly influenced by density, N application, variety, and density × variety interaction; however, no interaction effects of density × N rate and N rate × variety were observed in 2013 (**Table S2**). During three growing seasons, the highest total dry matter (TDM) accumulation for DH618 (26.4  Mg ha^−1^) and DH605 (30.3 Mg ha^−1^) were both obtained under HDN360 treatment in 2013. The average biomass of DH605 (23.9 Mg ha^−1^) was higher than that of DH618 (22.1 Mg ha^−1^) through three growing seasons. Above-ground biomass at tasseling and harvest both exhibited significant improvements under the HD condition compared to the LD condition in both varieties (**Figure 2**). For example, in 2013, the biomasses were 27.4% and 26.2% higher for DH618 and 34.5% and 30.9% higher for DH605 at tasseling and harvest, respectively. Moreover, the impacts of N on biomass differed through growing stages under different densities, and the enhancements were marginal at tasseling. Using DH618 in 2013 as an example, with the increasing N (N180, N360, N540 vs. N0), the biomass at tasseling stage increased 4.8%, 6.2%, 7.8% and 5.6%, 6.8%, 7.2% at LD and MD, respectively, while it increased 2.5%, 4.7%, and 6.7% at HD. Nevertheless, at harvest, the response to N became more intensive: the biomass increased approximately 9.3%, 12.5%, and 10.4% (LD); 11%, 16.3%, and 14.5% (MD); and 12.5%, 19.4%, and 13.1% (HD) at harvest in N180, N360, and N540 treatments compared to the N0, respectively.

### Leaf Area Index

**Figure 3:**
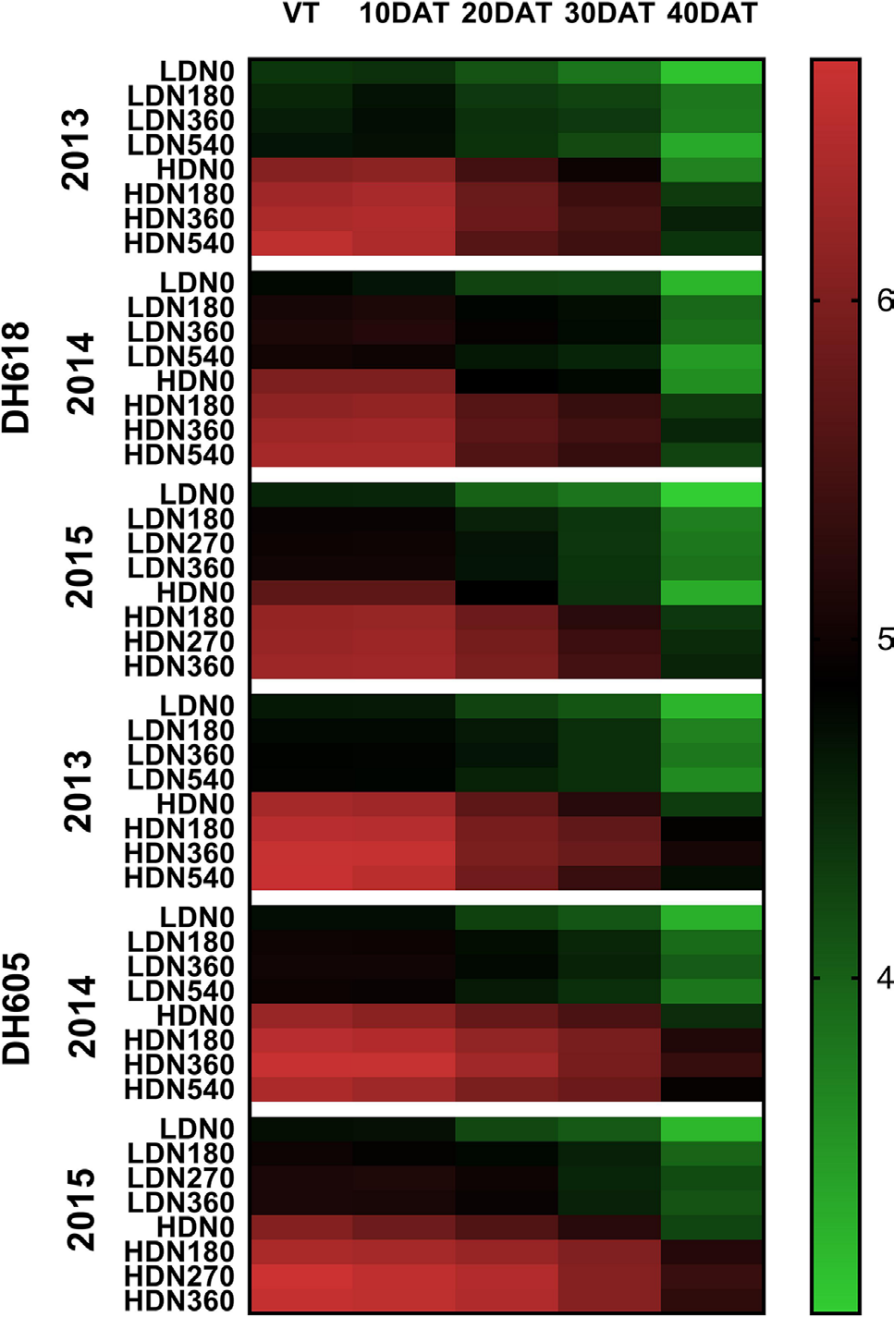
Variations in leaf area index as affected by densities and nitrogen application rates of DH618 and DH605 during the 2013–2015 growing seasons. VT, 10DAT, 20DAT, 30DAT, and 40DAT represent the tasseling stage and 10, 20, 30, and 40 days after tasseling, respectively. LD and HD refer to low density and high density; N0, N180, N270, N360, and N540 represent nitrogen rates of 0, 180, 270, 360, and 540 kg ha^−1^, respectively.

During the 2013–2015 growing seasons, LAI at each stage was influenced significantly by the density, N application, and variety (**Table S3**). However, factor interactions differed among years. For example, the density × N rate interaction was significant in 2013 and 2015, whereas this interaction effect was found only for LAI at 40DAT in 2014. Nevertheless, the density × N rate × variety three-way interaction for LAI at 30DAT and 40DAT was all significant among the three growing seasons. The LAI was commonly higher for DH605 than for DH618. For both varieties, the highest LAIs were observed at HD for each growing stage (**Figure 3**). From sowing to 40DAT (2013), DH618 and DH605 obtained an average of 39.5%, 36.8%, 31.4%, 28.3%, 21.6% and 37.0%, 36.5%, 29.6%, 27.1%, 32.7% higher LAI, respectively (HD vs. LD). Similarly, considerable differences among different N applications were found during the whole growing seasons. Within the tested variation of N levels, N180–N360 tended to be more effective than N540 in increasing and maintaining the LAI: N540 decreased 28.7% for LD and 33.2% for HD, whereas N360 decreased 18.3% and 28.6%, respectively from VT to 40DAT (DH618 in 2013). With the growth process moving forward, the response of LAI to N (N180, N360, and N540 vs. N0) under different density conditions increased. Taking DH618 in 2013 as an example, the LAI at the VT stage increased 2.9%, 4.8%, 6.5% and 3.9%, 5.6%, 8.5% for LD and HD, respectively. While at 40DAT, LAI increased 21.5%, 20.9%, 7.3% and 18.2%, 24.5%, 19.7% for LD and HD, respectively.

### Canopy Apparent Photosynthesis

**Figure 4:**
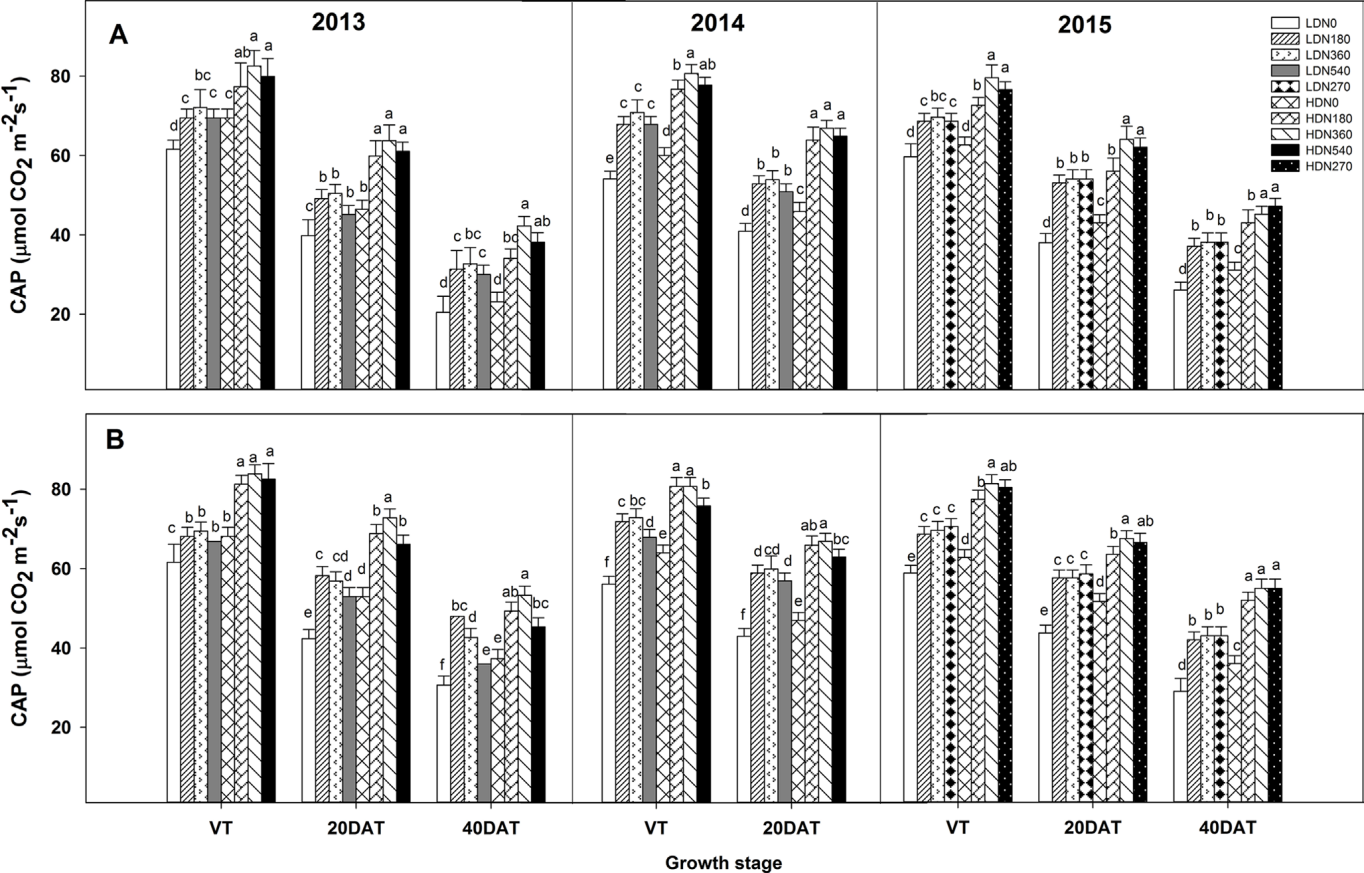
Effects of density and nitrogen application rate on the canopy apparent photosynthesis (CAP) of DH618 **(A)** and DH605 **(B)** during the 2013–2015 growing seasons. LD and HD refer to low density and high density; N0, N180, N270, N360, and N540 represent nitrogen rates of 0, 180, 270, 360, and 540 kg ha^−1^, respectively. VT, 20DAT, and 40DAT represent the tasseling stage and 20 and 40 days after tasseling, respectively. Different lowercase letters at the same growth stage indicate significant differences (*p* < 0.05) among the treatments.

During the three growing seasons, CAP was significantly influenced by density and N application, as well as by the density × N interaction; however, this interaction effect was not observed at the tasseling (VT) stage in 2013 and 2014 (**Table S4**). The CAP exhibited significant improvements in the HD condition compared to the LD condition for both varieties (**Figures 4** and **S3**; e.g., HD got an average 13.5%, 25.3%, and 20.2% higher CAP value than LD at VT, 20DAT, and 40DAT respectively for DH618 in 2013). Moreover, CAP responded intensively to N (N180 and N360 vs. N0) with increasing density. Using DH618 in 2013 as an example, the CAP increased approximately 27% and 37% at 20DAT and 60% and 82% at 40DAT under LD and HD conditions, respectively. The CAP under N540 was generally lower than that under N360 at these two density levels. Moreover, N540 hastened the decrease in CAP after tasseling, particularly under HD condition (e.g., a decrease in 53% from VT to 40DAT). Besides, in the absence of N, the CAP declined sharply when compared to that in plants applied with N (e.g., in 2013, N360 obtained a 18.9% higher CAP than N0 at HD, and the decline rates from VT to 40DAT were 66.6% and 48.8% for N0 and N360, respectively). The response of CAP to density was consistent between the two varieties; however, the response to N was different. For instance, the CAP for DH618 under LD was decreased compared to that in the control treatment (LDN360), whereas enhancements were found for DH605 under LDN180 during the 2013 and 2014 growing seasons.

### Enzyme Activity

**Figure 5:**
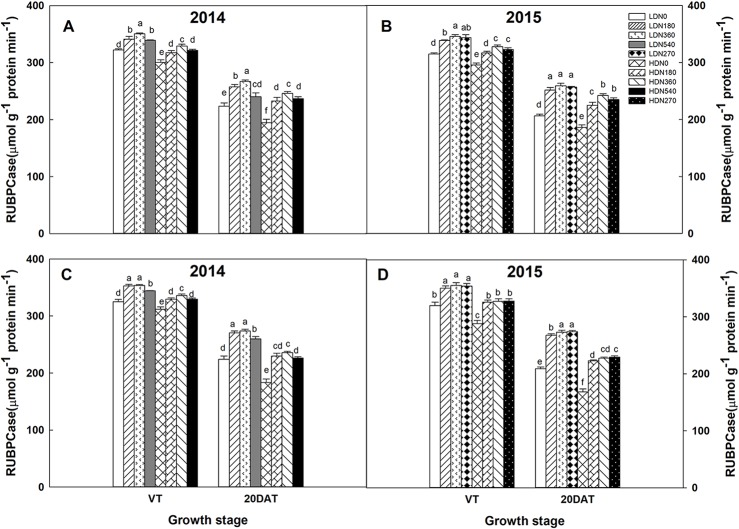
Effects of density and nitrogen application rate on ribulose-1,5-bisphosphate carboxylase (RuBPCase) activity in **(A**, **B)** DH618 and **(C**, **D)** DH605 during the 2014 and 2015 growing seasons. VT and 20DAT represent the tasseling stage and 20 days after tasseling, respectively. Different lowercase letters indicate a significant difference (*p* < 0.05) among treatments at the same growth stage. LD and HD refer to low density and high density, respectively; N0, N180, N270, N360, and N540 represent nitrogen rates of 0, 180, 270, 360, and 540 kg ha^−1^, respectively.

**Figure 6:**
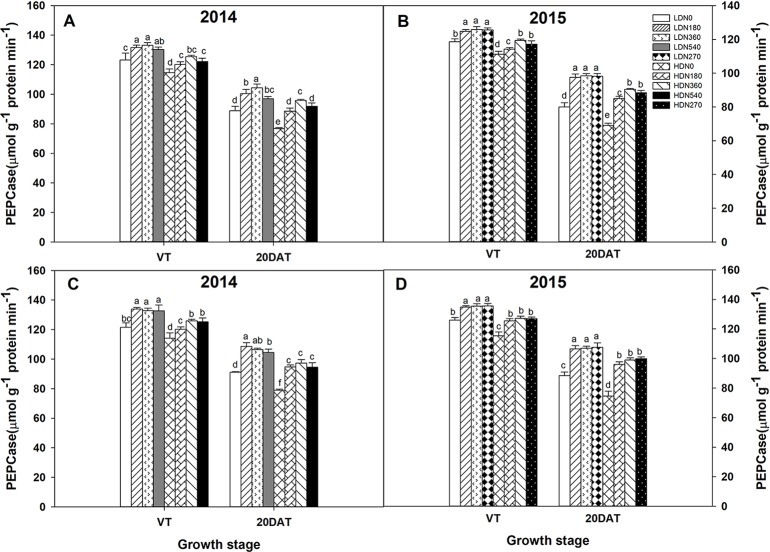
Effects of density and nitrogen application rate on phosphoenolpyruvate carboxylase (PEPCase) activity in **(A**, **B)** DH618 and **(C**, **D)** DH605 during the 2014 and 2015 growing seasons. VT and 20DAT represent the tasseling stage and 20 days after tasseling, respectively. Different lowercase letters indicate a significant difference (*p* < 0.05) among treatments at the same growth stage. LD and HD refer to low density and high density, respectively; N0, N180, N270, N360, and N540 represent nitrogen rates of 0, 180, 270, 360, and 540 kg ha^−1^, respectively.

During the 2014 and 2015 growing seasons, the activities of RuBPCase and PEPCase were significantly affected by planting density and N application rate; however, the interaction effects of density × N on the enzyme activities differed (**Table S5**). Increased stand density resulted in decreased activities of RuBPCase and PEPCase during both growing seasons (**Figures 5** and **6**), whereas an advance occurred with the increasing N application. Moreover, the responses to N levels were superior at HD versus LD. Using RuBPCase activities of DH618 (20DAT, 2015) as an example, enhancements of 22.0%, 24.5%, and 25.6% (LD) and 20.9%, 26.9%, and 30.1% (HD) were gained with N180, N270, and N360 treatments, respectively. The highest activities of RuBPCase and PEPCase were both detected for N360 rather than for the highest N rate (N540) at each density in 2014. In 2015, though N360 achieved the highest value at each stage for each enzyme activity, no significant difference was detected among the N levels from N180 to N360 at 20DAT under LD, and there was no significant difference between N270 and N360 under HD similarly. In the absence of N supply, the activities of both RuBPCase and PEPCase were restrained and sharply declined compared to those in plots receiving N. For example, the PEPCase activities of DH618 in the HD condition decreased 32.6% and 21.7% for N0 and N360, respectively, from VT to 20DAT in 2015.

### ^13^C-Photosynthate Proportional Allocation at Tasseling Stage

At 24 h after labeling, the ^13^C-photosynthate allocation ratios among different organs were affected significantly by density, N application rate, variety, and most of their interactions during the 2014–2015 growing seasons (**Table 3**). The ^13^C-photosynthate allocation was observed mainly in the stem, followed by other leaves and bract + cob for both varieties, while the distribution patterns were different. For example, the ^13^C-photosynthate allocation ratios in the stem, other leaves, ear leaves, tassel, and bract + cob were an average of 53.5%, 29.4%, 5.4%, 4.0%, and 7.7% and 55.2%, 28.4%, 4.5%, 2.8%, and 9.1% for DH618 and DH605, respectively, in 2015. Increased planting density was always accompanied by an increase in ^13^C-photosynthate allocation ratio to the stem, yet a decrease in allocation ratio to bract + cob. Take the data in 2015 as an example, the allocation ratio to stem increased 2.0% and 1.8% for DH618 and DH605, while the allocation ratio to bract + cob decreased 13.5% and 5.8%, respectively. Considerable differences among different N applications were also found, but the impacts of N on the ^13^C-photosynthate allocation ratio were opposite to those of density. Besides, the reactions of ^13^C-photosynthate allocation were different between varieties. For instance, in 2015, the ^13^C-photosynthate allocation ratio in stem decreased 2.4%, 3.6%, and 4.4% and 1.1%, 1.4%, and 1.6% for DH618 and DH605 (N180, N270, and N360 vs. N0), whereas the allocation ratio in bract + cob increased 13.0%, 17.3%, and 19.4% and 6.1%, 9.2%, and 8.7%, respectively.

**Table 3:** Effects of density and nitrogen application rate on ^13^C-photosynthate proportional allocation among above-ground plant organs (%) at the tasseling stage during the 2014 and 2015 growing seasons.

Factors		^13^C-photosynthate distribution in different organs (%)									
		2014					2015				
		Stem	Other leaves	Ear leaf	Tassel	Bract + Cob	Stem	Other leaves	Ear leaf	Tassel	Bract + Cob
DH618											
LD	N0	52.48b	30.81bc	4.55c	4.72c	7.43e	54.2b	29.11b	5.21f	4.16b	7.31f
	N180	51.02cd	30.84bc	4.35d	4.84b	8.96b	52.97cd	28.95b	5.41de	4.26a	8.41c
	N270			–			52.55cd	29.25ab	5.48bc	4.15b	8.57b
	N360	50.48e	30.93bc	4.41d	4.98a	9.21a	52.2d	29.39ab	5.54b	4.15b	8.73a
HD	N0	53.14a	30.49c	4.77b	4.57d	7.04f	55.69a	29.29ab	4.77g	3.87d	6.38h
	N180	51.46c	31.09b	4.56c	4.75c	8.14c	54.25b	29.32ab	5.39e	3.97c	7.08g
	N270			–			53.39c	29.80ab	5.47cd	3.86d	7.49e
	N360	50.77d	31.39a	5.02a	4.57d	8.24d	52.79cd	30.09a	5.65a	3.85d	7.62d
DH605											
LD	N0	54.18b	28.80a	4.19c	2.57a	10.26d	55.36c	28.55a	4.42e	2.84b	8.83e
	N180	52.44c	28.91a	4.21bc	2.46b	12.32b	54.69d	28.53a	4.60c	2.83b	9.35c
	N270			–			54.45d	28.32b	4.78b	2.80c	9.65a
	N360	51.91c	29.09a	4.65a	2.46b	12.98a	54.45d	28.48a	4.83a	2.71e	9.53b
HD	N0	56.30a	27.80b	4.02d	2.29c	9.60e	56.25a	28.31b	4.32f	2.83b	8.28f
	N180	54.83b	28.44ab	4.18c	2.21d	10.34d	55.73b	28.24b	4.43e	2.79c	8.81e
	N270			–			55.55bc	28.2b	4.44e	2.77d	9.04d
	N360	54.34b	28.59a	4.27b	2.26cd	10.54c	55.41bc	28.19b	4.47d	2.87a	9.06d
ANOVA											
Density (D)		109.4***	6.1*	31.2***	525.5***	3,543.2***	507.7***	12.4**	933.4***	1,578.3***	3,032.6**^*^
N rate (N)		85.1***	11.3***	137.6***	4.2*	2,079.2***	258.9***	14.2***	1,224.3***	84.6***	1,004.6***
Variety (V)		309.7***	583.6***	688.4***	59,389.4***	18,278.8***	1,464.8***	1,082.1***	22,690.6***	126,294.3***	8,308.7***
D × N		0 ns	4.9*	4.8*	13.8***	355.4***	4.7**	3.7*	45.6***	49***	7.7*^*^
D × V		42.2***	18.5***	403.3***	2.4 ns	372.8***	0.3 ns	117.4***	196.7***	2,092.8***	364.7***
N × V		0 ns	0.1 ns	48***	59.1***	63.9***	55.1***	28.5***	169.8***	63.7***	66.4**^*^
D × N × V		0.3 ns	0.5 ns	76.3***	33.2***	43.9***	6.1**	3.4*	260***	51.1***	8.8***

Different lowercase letters indicate a significant difference (p < 0.05) with the same organ of the same variety among treatments. The numbers in the ANOVA section represent F values. ns, not significant. *Significant at the 0.05 probability level. ***Significant at the 0.01 probability level. ***Significant at the 0.001 probability level.

### ^13^C-Photosynthate Proportional Allocation at Physiological Maturity Stage

The density, N rate, variety, and their interactions likewise altered the ^13^C-photosynthate distribution ratio distinctly at physiological maturity (**Table 4**). At R6, the distribution of ^13^C-photosynthate in grain (52.3–61.5%) was higher than that to other organs, followed by stem (16.1–21.9%). The ^13^C-photosynthate allocation ratio to grain was always reduced in HD compared to LD, whereas the ratios to stem and other leaves were increased in both experimental years (2014 and 2015). In 2015, the allocation ratio to grain decreased 3.7% and 3.4% for DH618 and DH605, while the allocation ratio to stem increased for 6.2% and 7.5%, respectively. Increased N application caused a considerable increase in ^13^C-photosynthate allocation ratio to grain but decreased the allocation ratio to stem, other leaves, ear leaves, and tassel. Within the tested variation of N levels, N180 and N270 tended to be more effective than N360; nevertheless, the response of ^13^C-photosynthate allocation was different between varieties. For instance, in 2015, the ^13^C-photosynthate allocation ratio in grain decreased 9.6%, 9.8%, and 8.8% for DH618 (N180, N270, and N360 vs. N0), whereas the allocation ratio for DH605 only increased for 5.7%, 6.4%, and 5.8%, respectively. Besides, the N effect was enlarged under the HD condition. As compared to that for N0, the ^13^C-photosynthate distribution rate in grain was 9.7%, 9.0%, and 7.7% and 10.1%, 9.8%, and 9.9% higher at LD and HD, respectively, for N180, N270, and N360 in the 2015 growing season for DH618.

**Table 4:** Effects of density and nitrogen application rate on 13C-photosynthate proportional allocation among the above-ground plant organs (%) at maturity stage during the 2014 and 2015 growing seasons.

Factors		^13^C-photosynthate distribution in different organs (%)											
		2014						2015					
		Stem	Other leaves	Ear leaf	Tassel	Bract + Cob	Grain	Stem	Other leaves	Ear leaf	Tassel	Bract + Cob	Grain
DH618													
LD	N0	19.27b	11.55b	2.16a	1.00b	10.67d	55.34d	20.65b	11.74b	2.13c	1.11c	10.69g	53.68f
	N180	16.07e	9.03e	1.76c	0.72f	10.95c	61.47a	17.06e	10.29e	1.67ef	1.22a	10.88f	58.88a
	N270				–			17.15e	10.50d	1.52g	1.13c	11.18e	58.53b
	N360	16.72d	9.53d	1.83b	0.73e	11.5a	59.69b	17.60d	9.88f	2.19b	1.19b	11.35d	57.81c
HD	N0	21.83a	11.84a	2.16a	1.12a	10.1e	52.93e	21.93a	12.20a	2.24a	0.9d	11.45cd	51.28g
	N180	17.75c	10.38c	1.70d	0.87c	10.98c	58.33c	18.64c	11.00c	1.76d	0.67e	11.53c	56.39e
	N270				–			18.27d	10.88	1.70e	0.67e	11.97b	56.52d
	N360	17.53c	10.59c	1.54e	0.78d	11.28b	58.28c	18.14d	10.90c	1.65f	0.64e	12.3a	56.36e
DH605													
LD	N0	20.25b	10.66b	1.88a	0.90c	13.46b	52.85c	19.17c	10.08b	1.9b	0.95b	11.96a	55.94e
	N180	17.31e	9.41d	1.80abc	0.78d	12.88e	57.82a	17.61g	9.50g	1.57f	0.8f	11.58c	58.95a
	N270			–				17.86f	9.48g	1.55e	0.82f	11.48cd	58.81a
	N360	17.34e	9.63d	1.77bc	0.79d	13.34c	57.13a	17.97f	9.61f	1.53e	0.82g	11.71b	58.36b
HD	N0	21.16a	11.56a	1.88a	0.94a	13.79a	50.67d	20.92a	10.85a	2.22a	1.05a	11.70b	53.26f
	N180	19.61c	10.11c	1.83ab	0.91b	13.12d	54.41b	19.39b	9.93c	1.74d	0.88e	11.57c	56.48d
	N270			–				18.77e	9.82d	1.76c	0.89d	11.44d	57.31c
	N360	19.25d	10.01c	1.73c	0.89c	13.31c	54.82b	18.95d	9.69e	1.79d	0.88c	11.55cd	57.14c
ANOVA													
Density (D)		916.4***	184.5***	23.4***	2,328.9***	2.8 ns	174.1***	2,932.0***	629.3***	375.8***	2,226.2***	419.5***	3091***
N rate (N)		1221***	332***	231.8***	3,762.1***	132.2***	292.5***	3,085.4***	698.1***	1,841.4***	227.2***	70.9***	3,065.3***
Variety (V)		293.5***	20.2***	11.6**	2.8 ns	1,2841.3***	264.9***	42.4***	2515***	382.1***	195.8***	102.0***	565.0***
D × N		10.8***	4.9*	18.2***	92.1***	16.5***	5.0*	87.2***	7.1**	247.3***	122.8***	7.5**	59.0***
D × V		0 ns	4.4*	20***	7.7*	103.4***	0.7 ns	23.4***	32.6***	747.2***	4,566.2***	731.2***	5.5*
N × V		56.2***	10.4**	91.9***	1,294.8***	389.3***	4.7*	409.3**	61.1*	161.5***	30.3***	178.8***	139.8***
D × N × V		58.2***	13.7***	8.2**	88.2***	28.9***	0.8 ns	11.6***	39.8***	291.7***	92.7***	8.4***	8.0***

Different lowercase letters indicate a significant difference (p < 0.05) with the same organ of the same variety among treatments. The numbers in the ANOVA section represent F values. ns, not significant. *Significant at the 0.05 probability level.**Significant at the 0.01 probability level. ***Significant at the 0.001 probability level.

### Correlation Analysis

**Figure 7:**
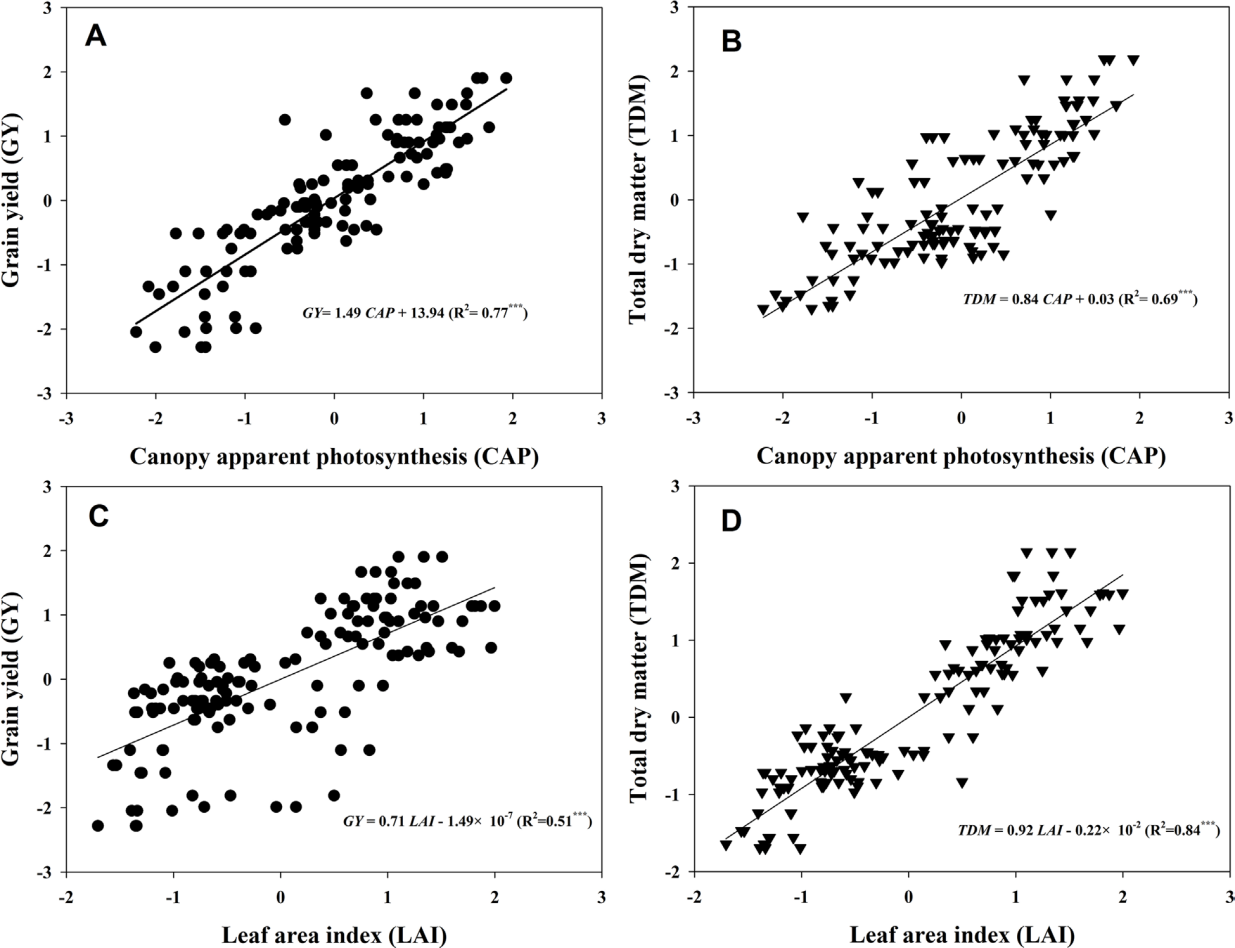
Relationships between canopy apparent photosynthesis (CAP), leaf area index (LAI), grain yield (GY), and total dry matter (TDM). The data shown in the figure were the standardized GY, TDM, CAP, and LAI values of the two varieties grown at low and high densities with different levels of nitrogen during 2013–2015. The standardized LAI values at tasseling and 20 and 40 days after tasseling were selected in accordance with the CAP data to fit the linear relationships between CAP, LAI, GY, and TDM (*n* = 128). Asterisks represent significance at the 0.001 probability level.

**Figure 8:**
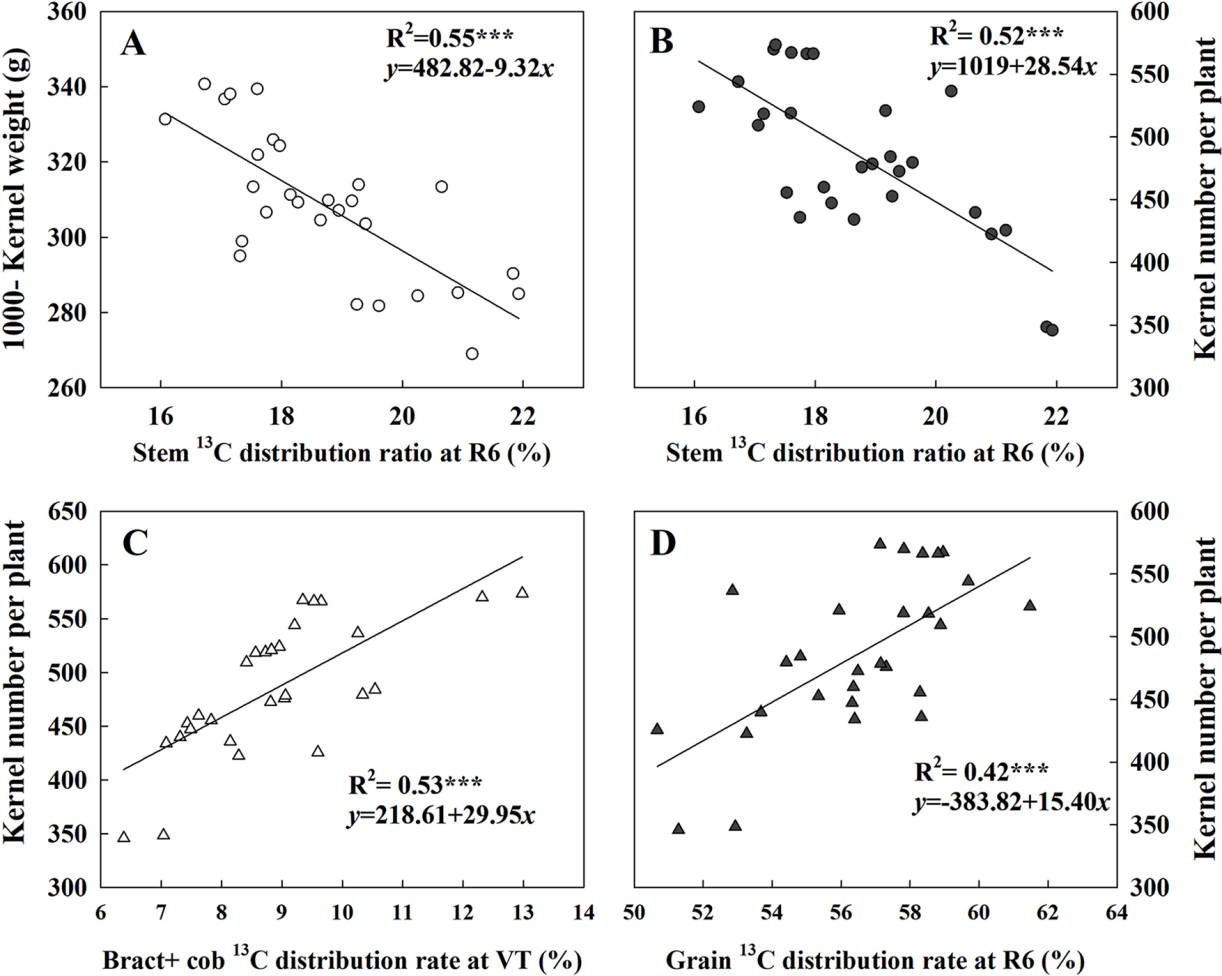
Relationships between ^13^C-photosynthate allocation ratio (%) in different organs and 1,000-kernel weight and kernel number. Data correspond to plants grown at low and high densities with different levels of nitrogen. The correlation coefficients (*R*^2^) were calculated. ***Significance at the 0.001 probability level (*n* = 28).

**Figure 7** presented the linear fitting results between CAP, LAI, GY, and TDM over the three experimental years. The CAP was more related to GY (*R*^2^ = 0.77^***^) than LAI (*R*^2^ = 0.51^***^), while LAI was more related to TDM (*R*^2^ = 0.84^***^) rather than GY (*R*^2^ = 0.69^***^). The regression equations were *GY* = 1.49*CAP* + 13.94, *TDM* = 0.84*CAP* + 0.03, *GY* = 0.71*LAI* − 1.49 × 10^−7^, and *TDM* = 0.92*LAI* − 0.22 × 10^−2^. Both TKW and KNP exhibited negative relationships with ^13^C-photosynthate distribution to stem at R6 (*R*^2^ = 0.55^***^ and 0.52^***^) (**Figures 8A, B**). Meanwhile, positive correlations were found between KNP and ^13^C-photosynthate distribution ratio to bract + cob at VT (*R*^2^ = 0.53^***^) and to grain at R6 (*R*^2^ = 0.42^***^) (**Figures 8C, D**).

## Discussion

In this study, the highest yield of 17.1 Mg ha^−1^ was achieved at a density of 9.75 pl m^−2^ combined with a N supply of 360 kg ha^−1^. For comparison, Testa et al. (2016) obtained a lower GY (16.9 Mg ha^−1^) under the 11.25 pl m^−2^ density condition, whereas Ciampitti et al. (2013c) reported GY was maximized (13.6 Mg ha^−1^) at a density of 7.9 pl m^−2^ and N rate of 224 kg ha^−1^. Maize yield is population reliant (Zhao et al., 1997); its low per-plant yield potential renders high densities imperative. However, increasing density beyond the optimal level is not the remedy, which may most probably cause GY decrease due to interplant competition for light and nutrients and restrain kernel setting and filling, as reflected by the results of density 11.25 pl m^−2^ in our research (2014). Nevertheless, stalk lodging is not the limiting factor affecting GY under extra-HD, and this may be due to the lodging-resistant trait of the testing varieties; lodging occurred only in 2014 for DH605 at HD because of different weather conditions. At HD conditions, crops required greater N fertilizer to offset the per-plant lower N availability. Similarly, excess N supply did not result in a GY advantage in maize, particularly at LD in this study, which was in agreement with most previous studies (Tollenaar and Lee, 2002; Smith et al., 2004; Jin et al., 2012). Although numerous studies have focused on optimizing planting density and N application rate, it remains a difficult issue due to strong genotype × density × N interactions (Ciampitti and Vyn, 2012).

High GY depends on an optimum canopy structure, which requires an adequate LAI to enable source supply (Wei et al., 2017). Previous studies have shown high LAI was associated with proper high planting density and N rate (Olsen and Weiner, 2007; Xu et al., 2017). This supported the current finding that HD combined with an N rate of 180–360 kg ha^−1^ obtained a top LAI and maintained a longer duration of high LAI (**Figure 4**). Longer LAI duration is important for high GY; hence, we also attributed the higher LAI at 40DAT to the “stay green” trait of both varieties.

Actually, the improved leaf photosynthetic capacity does provide a basic solution for enhancing maize yield (Ren et al., 2016). In the current study, increased stand density and N rate both resulted in expected enhancements in CAP (**Figure 4**), which was consistent with previous findings (Yang et al., 2010; Wei et al., 2011). Nevertheless, the response of CAP to density is more intensive in comparison to its response to N rate for both varieties, which indicated density had a better performance in regulating CAP. A tight relationship between LAI and CAP was found in our study (**Figure S1**; *R*^2^ = 0.71^***^), as previous observations reported (Dong et al., 2000; Liu et al., 2015). However, the degradation in CAP cannot always explained by the decrement in LAI, as the descent rate observed in LAI is much lower compared to CAP from VT to 40DAT (**Figures 3** and **5**). Actually, under field conditions, the photosynthetic potential is also dependent upon the photosynthetic physiological function in the interior of green leaves. The degradation of RuBPCase and PEPCase has been demonstrated to cause decreased photosynthetic capacity during leaf senescence (Crafts-Brandner and Saluki, 2000; Ding et al., 2005). Hence, the higher RuBPCase and PEPCase activities detected under N360 could partly account for its larger CAP compared to other N rates under each density (**Figure 3**). Similarly, we also prefer attributing the CAP degradation to the relative rapid drops in RuBPCase and PEPCase activities (**Figures 6** and **7**), in agreement with Ding et al. (2005).

The relationships between LAI, CAP, and GY were evaluated. As expected, close links between LAI and GY (**Figure 8A**, *R*^2^ = 0.51^***^) and CAP and GY (**Figure 8B**, *R*^2^ = 0.77^***^) were observed. Apparently, increments in GY with increasing density and N rate were, in most cases, more associated with increases in CAP than with increments in LAI. However, the LAI seemed to be more related to TDM. This result suggested that larger LAI does not always mean higher GY, but the total biomass could still be predicted by LAI, according to their tight correlation (*R*^2^ = 0.84^***^). In addition, a close correlation between CAP and KNA was found (**Figure S2**), which was consistent with a previous study (Edmeades and Daynard, 1979), although the curvilinear response of kernel number to photosynthesis during the critical period around flowering was established in single plant levels. Hence, we infer that maize canopy displaying a higher capacity of CAP (especially at VT) may usually acquire a greater GY by producing more kernels, as kernel setting is particularly sensitive to resource competition (Borras and Vitantonio-Mazzini, 2018).

GY depended on not only the photosynthesis of leaves but also the subsequent biomass allocation to ear for kernel setting and kernel filling (Allison, 2008; Ning et al., 2013), as kernel number and KW are key factors affecting GY (Rossini et al., 2011; Ordóñez et al., 2015). The responses of KNP to plant growth rate during the critical period for kernel setting as affected by density and nitrogen rate have been well studied (Tollenaar et al., 1992; Andrade et al., 2002). Our current results showed that exacerbated interplant competition induced by density increase or N deficiency both reduced the labeled ^13^C assimilates allocation ratio in the reproductive organ at both the VT and R6 stages. Besides, we identified notable responses of KNP to ^13^C-photosynthate distribution to reproductive organs (**Figure 8**). Hence, the significantly decreased KNP under the HD or N0 condition could be explained, partly by the reduction in assimilate reallocation to ear. Moreover, the higher correlation between KNP and ^13^C-photosynthate allocation ratio to ear (bract + cob) at VT indicated that an improved ^13^C allocation ratio to ear is crucial for kernel setting. Besides, a previous study has shown that genotypic differences in the response of KNP to plant growth rate were related to the effects of N on biomass partitioning to the ear (D’Andrea et al., 2008). Here, we attribute the higher KNP obtained by DH605 to its high reallocation ratio to ear at tasseling stage.

In this research, the labeled ^13^CO_2_ was applied at tasseling stage, and most of it accumulated in nonear organs 24 h later (**Table 3**). Consequently, data at R6 tend to be more useful for demonstrating the use of reserves in maize GY determination among treatments (**Table 4**). Interestingly, the allocation of ^13^C-photosynthate to grain at R6 in 2014 showed a distinctive pattern compared to CAP and LAI; it decreased when N rates were higher than 180 kg ha^−1^ under LD condition and was hardly changed even when N rate increased under the HD condition. These results indicated that both N deficiency and N excessiveness could restrain the reallocation of ^13^C-photosynthate to grain. Moreover, the negative relationship between KNP and TKW with ^13^C-photosynthate allocation ratio to stem (R6) illustrated that restraining the transfer of ^13^C-photosynthate from stems to ears could impair both KNP and TKW and result in reduced GY. In the present study, though the highest ^13^C-photosynthate allocation ratio to grain was observed under LDN180 treatment, this still could not compensate the yield loss caused by the small population. Moreover, the significant correlation between CAP and KNA also suggested the important role of assimilation ability in kernel number formation. Overall, strong assimilation ability combined with high retransfer ability is quite important for further improvement in KNP and TKW.

In the current study, HD (9.75 pl m^−2^) in combination with 180–360 kg N ha^−1^ resulted in considerable improvement in maize GY. Maize GY, biomass, CAP, LAI, and ^13^C-photosynthate reallocation all responded more intensively to density than N rate, but the N response differed between two varieties. The negative effect of N deficiency or excessiveness on yield formation was evident. Elevated CAP appeared to be not only the result of high LAI but also contributed by better maintenance of enzyme activities involved in photosynthesis. Besides, relationships among CAP, LAI, biomass, GY, and KNA were established. HD increased the retention of ^13^C-photosynthate in vegetative organs and reduced the percentage of labeled ^13^C assimilates in ear, while N supply moderated the response. KNP was closely correlated to ^13^C-photosynthate in ears, and both KNP and TKW were negatively correlated to ^13^C assimilate distribution rate in stems at R6. Based on our results, maize plants with greater CAP, more ^13^C-photosynthate distribution to ears, and higher use of reserves under different density and N rate combinations could enhance KNA and achieve a higher GY consequently.

## Data Availability

The raw data supporting the conclusions of this manuscript will be made available by the authors, without undue reservation, to any qualified researcher.

## Ethics Statement

The varieties used in this study are high-yield and density-tolerance variety grown extensively in North China. The maize seeds were brought from Shandong Denghai Seeds Co., Ltd. As our experiment involves neither transgenic materials nor technology, it does not require ethical approval. The experimental research on plants performed in this study complies with institutional, national and international guidelines. The field study was conducted in accordance with local legislation.

## Author Contributions

SW designed the work, carried out all experiments and data analysis, and drafted the manuscript. XW helped perform field experiments and draft the manuscript. SD conceived the study and planned the experiments. GL helped draft the manuscript. DJ helped draft and revise the manuscript. All authors read and approved the final manuscript.

## Funding

This work was supported by the National Natural Science Foundation of China (nos. 31801302 and 31171497); the Postdoctoral Science Foundation funded project of China (no. 2017M611832); the Postdoctoral Science Foundation funded project of Jiangsu Province (no. 1701040A); the National Key Research and Development Program of China (no. 2016YFD0300308); the Natural Science Foundation of Jiangsu Province (no. BK20170720); the National Basic Research Program of China (no. 2011CB100105); the National Food Science and Technology of High-yield Program of China (no. 2011BAD16B09); and the 111 Project (no. B16026).

## Conflict of Interest Statement

The authors declare that the research was conducted in the absence of any commercial or financial relationships that could be construed as a potential conflict of interest.

## Abbreviations

CAP, canopy apparent photosynthesis; CR, canopy respiratory rate; LAI, leaf area index; RuBPCase, ribulose-1,5-bisphosphate carboxylase; PEPCase, phosphoenolpyruvate carboxylase; GY, grain yield; KNP, kernel number per plant; KNA, kernel number per unit area (m^−2^); TKW, 1,000-kernel weight; VT, tasseling stage; DAT, days after tasseling; R6, physiological maturity.

## Supplementary Material

Supplementary Table 1 Effects of density and N rate on kernel number and 1000-kernel weight of DH618 and DH605 during the 2013 to 2015 growing seasons.

Supplementary Table 2 Analysis of variance of dry matter accumulation as affected by density, N rate, and variety.

Supplementary Table 3 Analysis of variance of leaf area index (LAI) at different growth stages as affected by density, N rate, and variety.

Supplementary Table 4 Analysis of variance of canopy apparent photosynthesis (CAP) at different growth stages as affected by density, N rate, and variety.

Supplementary Table 5 Analysis of variance of RUBPCase and PEPCase activities at different growth stages as affected by density, N rate and variety.

Supplementary Figure S1 Relationship between leaf area index (LAI) and canopy apparent photosynthesis (CAP). The data shown in figure were LAI and CAP values of the two varieties grown at low and high densities with different levels of nitrogen during 2013–2015 (n = 128). Asterisks (***) represent significance at the 0.001-probability level.

Supplementary Figure S2 Relationship between canopy apparent photosynthesis (CAP) and kernel number per unit area (KNA). The data shown in the figure was the standardized CAP and KNA values of the two varieties grown at low and high densities with different levels of nitrogen during 2013–2015 (n = 128). Asterisks (***) represent significance at the 0.001-probability level.

Supplementary Figure S3 The relative variation ratio of canopy apparent photosynthesis (CAP, %) under different density and nitrogen application rate combinations compared to LDN360 of DH618 **(A)** and DH605 **(B)** during the 2013–2015 growing seasons. LD and HD refer to low density and high density; N0, N180, N270, N360, and N540 represent nitrogen rates of 0, 180, 270, 360, and 540 kg ha^–1^, respectively VT, 20DAT, and 40DAT represent the tasseling stage, and 20 nd 40 days after tasseling, respectively.
