# Supplementary material for: Maize Canopy Apparent Photosynthesis and 13C-Photosynthate Reallocation in Response to Different Density and N Rate Combinations
Source: Front Plant Sci. 2019 Sep 19;10:1113. doi: 10.3389/fpls.2019.01113 (PMC6761910; doi:10.3389/fpls.2019.01113)
Supplement: Supplementary Table 1 — Effects of density and N rate on kernel number and 1000-kernel weight of DH618 and DH605 during the 2013 to 2015 growing seasons. [file Table_1.doc]

Supplementary Material

**Maize canopy apparent photosynthesis and ^13^C-photosynthate reallocation in response to different density and N rate combinations**

**Shanshan Wei****^1, 2^, Xiangyu Wang^2, 3^, Guanghao Li^2^, Dong Jiang^1, *^, Shuting Dong^2, *^**

***Correspondence:** Dong Jiang ([jiangd@njau.edu.cn](mailto:jiangd@njau.edu.cn))**;** Shuting Dong ([stdong@sdau.edu.cn](mailto:stdong@sdau.edu.cn))

**Supplementary Table 1** Effects of density and N rate on kernel number and 1000-kernel weight of DH618 and DH605 during the 2013 to 2015 growing seasons.

| **Density** | **N rate** | **Kernel number (n.m^-2^)** | | | | | | **Kernel number (n.pl^-1^)** | | | | | | **1000-Kernels weight (g)** | | | | | |
| --- | --- | --- | --- | --- | --- | --- | --- | --- | --- | --- | --- | --- | --- | --- | --- | --- | --- | --- | --- |
|  |  | **2013** | | **2014** | | **2015** | | **2013** | | **2014** | | **2015** | | **2013** | | **2014** | | **2015** | |
|  |  | **DH618** | **DH605** | **DH618** | **DH605** | **DH618** | **DH605** | **DH618** | **DH605** | **DH618** | **DH605** | **DH618** | **DH605** | **DH618** | **DH605** | **DH618** | **DH605** | **DH618** | **DH605** |
| **LD** | **N0** | **2996f** | **3262ef** | **2804g** | **3381d** | **2707e** | **3283c** | **478.5b** | **519.3bc** | **452.6e** | **536.5b** | **439.7bc** | **520.9b** | **327.8f** | **335.7b** | **314.0d** | **284.4de** | **313.4b** | **309.7c** |
|  | **N180** | **3315de** | **3500d** | **3494d** | **3754c** | **3334c** | **3737b** | **520.2a** | **546.8a** | **524.0ab** | **569.8a** | **509.3a** | **567.2a** | **344.2b** | **345.4a** | **331.4b** | **295.0b** | **336.7a** | **321.9b** |
|  | **N270** | **-** | **-** | **-** | **-** | **3312c** | **3731b** | **-** | **-** | **-** | **-** | **518.9a** | **566.2a** | **-** | **-** | **-** | **-** | **338.1a** | **325.9ab** |
|  | **N360** | **3276e** | **3404de** | **3421de** | **3718c** | **3328c** | **3730b** | **515.3a** | **537.8ab** | **544.0a** | **573.4a** | **518.2a** | **566.3a** | **350.0a** | **346a** | **340.7a** | **298.9a** | **339.4a** | **324.3a** |
|  | **N540** | **3250e** | **3385de** | **3298def** | **3752c** | **-** | **-** | **515.6a** | **537ab** | **515.4bc** | **570.7a** | **-** | **-** | **339.7cd** | **345a** | **334.4b** | **288cd** | **-** | **-** |
| **MD** | **N0** | **3362de** | **3475f** | **3089f** | **3668d** | **-** | **-** | **450.8bc** | **468.8e** | **401.1f** | **482.0c** | **-** | **-** | **317.9g** | **326.4c** | **300.8f** | **279.4f** | **-** | **-** |
|  | **N180** | **3707bc** | **3987c** | **3796c** | **4163c** |  |  | **480.1b** | **516.3bc** | **484.5d** | **531.3b** |  |  | **340cd** | **334.1b** | **315.9cd** | **289.4c** |  |  |
|  | **N360** | **3691bc** | **3947c** | **3852c** | **4148c** |  |  | **480.6b** | **512.3bc** | **493.8cd** | **533.2b** |  |  | **344.5b** | **334.5b** | **318.3c** | **289.2c** |  |  |
|  | **N540** | **3644c** | **3897c** | **3809c** | **4097c** |  |  | **478.0b** | **511.2bc** | **491.1cd** | **528.4b** |  |  | **334.9e** | **332.6b** | **311.9d** | **287.7cd** |  |  |
| **HD** | **N0** | **3555cd** | **3725b** | **3152f** | **3759b** | **3104d** | **3732b** | **407.0d** | **419.2f** | **348.4h** | **425.6d** | **345.9d** | **422.5d** | **309.5h** | **309.8d** | **290.4h** | **269g** | **284.9d** | **285.3e** |
|  | **N180** | **4014a** | **4280a** | **4121ab** | **4385a** | **4100b** | **4284a** | **442.7c** | **479.0de** | **435.8e** | **479.5c** | **434.2c** | **472.5c** | **337.5de** | **323.9c** | **306.7e** | **281.8ef** | **304.6c** | **303.6d** |
|  | **N270** | **-** | **-** | **-** | **-** | **4216ab** | **4339a** | **-** | **-** | **-** | **-** | **447.2bc** | **475.8c** | **-** | **-** | **-** | **-** | **309.3b** | **309.8cd** |
|  | **N360** | **4112a** | **4403a** | **4271a** | **4408a** | **4332a** | **4314a** | **455.5bc** | **497.4cd** | **455.5e** | **484.1c** | **459.8bc** | **478.5c** | **342.3bc** | **327.7c** | **313.4d** | **282.1ef** | **311.3b** | **307.1c** |
|  | **N540** | **3931ab** | **4038b** | **4232a** | **4352a** | **-** | **-** | **459.8bc** | **496.0cd** | **456.9e** | **480.0c** | **-** | **-** | **335.7e** | **325.3c** | **300.7f** | **282ef** | **-** | **-** |
| **extra-HD** | **N0** | **-** | **-** | **3234ef** | **3847b** | **-** | **-** | **-** | **-** | **320.1i** | **388.3e** | **-** | **-** | **-** | **-** | **278.3i** | **248.7i** | **-** | **-** |
|  | **N180** |  |  | **3926bc** | **4322a** |  |  |  |  | **375.3g** | **418.8d** |  |  |  |  | **289.9h** | **260.8h** |  |  |
|  | **N360** |  |  | **4116ab** | **4402a** |  |  |  |  | **396.0fg** | **426.6d** |  |  |  |  | **294.3g** | **263.4h** |  |  |
|  | **N540** |  |  | **4105ab** | **4408a** |  |  |  |  | **401.9f** | **426.2d** |  |  |  |  | **294.8g** | **262.9h** |  |  |
| **ANOVA** | |  | |  |  |  |  |  | |  |  |  |  |  | |  |  |  |  |
| **Density (D)** | | **174***** | | **45.3***** | | **454.5***** | | **84.9***** | | **395.1***** | | **454.5***** | | **327.9***** | | **1165.9***** | | **1228.5***** | |
| **N rate (N)** | | **38***** | | **127.6***** | | **78***** | | **26.9***** | | **1110.2***** | | **77.9***** | | **336.6***** | | **264.2***** | | **239.2***** | |
| **Variety (V)** | | **42.9***** | | **287.8***** | | **159.2***** | | **54.6***** | | **237.9***** | | **159.2***** | | **40.9***** | | **4275.6***** | | **82.2***** | |
| **D×N** | | **2.7*** | | **2.6**** | | **1.5ns** | | **1.9ns** | | **1.4ns** | | **1.5ns** | | **9.1***** | | **4.5***** | | **2.0ns** | |
| **D×V** | | **0.2ns** | | **93.2***** | | **5.3*** | | **0.1ns** | | **0.9ns** | | **5.3*** | | **52.6***** | | **52.7***** | | **56.5***** | |
| **N×V** | | **0.3ns** | | **102.5***** | | **6.8***** | | **0.3ns** | | **11.5***** | | **6.8***** | | **43.7***** | | **12.7***** | | **6.1**** | |
| **D×N×V** | | **0.6ns** | | **2.3*** | | **0.4ns** | | **0.7ns** | | **0.4ns** | | **0.4ns** | | **1.8ns** | | **5.4***** | | **2.5ns** | |

Note: Different lowercase letters indicate a significant difference (p < 0.05) with the same year of the same variety among treatments. The numbers in the ANOVA section represent F values. ^ns^ Not significant; ^*^ Significant at the 0.05 probability level; ^**^ Significant at the 0.01 probability level; ^***^ Significant at the 0.001 probability level. LD, MD, HD, and extra-HD refer to low density, medium density, high density, and extra-high density, respectively. N0, N180, N270, N360, and N540 represent nitrogen rates of 0, 180, 270, 360, and 540 kg ha^-1^, respectively.
