# Supplementary material for: Maize Canopy Apparent Photosynthesis and 13C-Photosynthate Reallocation in Response to Different Density and N Rate Combinations
Source: Front Plant Sci. 2019 Sep 19;10:1113. doi: 10.3389/fpls.2019.01113 (PMC6761910; doi:10.3389/fpls.2019.01113)
Supplement: Supplementary Table 2 — Analysis of variance of dry matter accumulation as affected by density, N rate, and variety. [file Table_2.doc]

Supplementary Material

**Maize canopy apparent photosynthesis and ^13^C-photosynthate reallocation in response to different density and N rate combinations**

**Shanshan Wei****^1, 2^, Xiangyu Wang^2, 3^, Guanghao Li^2^, Dong Jiang^1, *^, Shuting Dong^2, *^**

***Correspondence:** Dong Jiang ([jiangd@njau.edu.cn](mailto:jiangd@njau.edu.cn))**;** Shuting Dong ([stdong@sdau.edu.cn](mailto:stdong@sdau.edu.cn))

**Supplementary Table 2** Analysis of variance of dry matter accumulation as affected by density, N rate, and variety.

| Factor | Dry matter accumulation at VT | | | Total dry matter accumulation | | |
| --- | --- | --- | --- | --- | --- | --- |
|  | 2013 | 2014 | 2015 | 2013 | 2014 | 2015 |
| ANOVA |  |  |  |  |  |  |
| Density (D) | 312.4*** | 1501.3*** | 3044.4*** | 231.5*** | 2475*** | 4907.5*** |
| N rate (N) | 7.5*** | 146*** | 55.3*** | 29.4*** | 1780.8*** | 518.4*** |
| Variety (V) | 96.1*** | 468.8*** | 115.6*** | 113.1*** | 201.5*** | 315.2*** |
| D×N | 0.2ns | 9*** | 5.4** | 1.3ns | 28.4*** | 17.8*** |
| D×V | 13.8** | 6.5** | 8.3** | 7.8** | 147.6*** | 65.8*** |
| N×V | 0.1ns | 8.8*** | 3.8* | 0.1ns | 32.3*** | 5.2** |
| D×N×V | 0ns | 1.2ns | 0.2ns | 0.3ns | 3.4** | 0.6ns |

Note: ^ns^ Not significant; ^*^ Significant at the 0.05 probability level; ^**^ Significant at the 0.01 probability level; ^***^ Significant at the 0.001 probability level.
