# Supplementary material for: Maize Canopy Apparent Photosynthesis and 13C-Photosynthate Reallocation in Response to Different Density and N Rate Combinations
Source: Front Plant Sci. 2019 Sep 19;10:1113. doi: 10.3389/fpls.2019.01113 (PMC6761910; doi:10.3389/fpls.2019.01113)
Supplement: Supplementary Table 3 — Analysis of variance of leaf area index (LAI) at different growth stages as affected by density, N rate, and variety. [file Table_3.doc]

Supplementary Material

**Canopy apparent photosynthesis and ^13^C-photosynthate reallocation are vital to maize yield formation under different density and N rate combinations**

**Shanshan Wei****^1, 2^, Xiangyu Wang^2, 3^, Guanghao Li^2^, Dong Jiang^1, *^, Shuting Dong^2, *^**

***Correspondence:** Dong Jiang ([jiangd@njau.edu.cn](mailto:jiangd@njau.edu.cn))**;** Shuting Dong ([stdong@sdau.edu.cn](mailto:stdong@sdau.edu.cn))

**Supplementary Table 3** Analysis of variance of leaf area index (LAI) at different growth stages as affected by density, N rate, and variety.

| Year | Variation | Leaf area index (LAI) | | | | |
| --- | --- | --- | --- | --- | --- | --- |
|  |  | VT | 10 DAT | 20 DAT | 30 DAT | 40 DAT |
| 2013 | ANOVA |  |  |  |  |  |
|  | Density (D) | 6977.2*** | 6950.1*** | 7732.9*** | 2774.7*** | 2070.5*** |
|  | N rate (N) | 42.6*** | 40.5*** | 96.8*** | 100*** | 216.6*** |
|  | Variety (V) | 105.5*** | 42.5*** | 171.4*** | 83.5*** | 198.1*** |
|  | D×N | 3.3* | 0.6ns | 3.1* | 3.2* | 8.9*** |
|  | D×V | 0.3ns | 0.4ns | 0.3ns | 0ns | 98.6*** |
|  | N×V | 2.7ns | 1ns | 0.1ns | 3.5* | 2.5ns |
|  | D×N×V | 0.3ns | 0.5ns | 4.9** | 6.4** | 9.3*** |
| 2014 | ANOVA |  |  |  |  |  |
|  | Density (D) | 3752.1*** | 4192.4*** | 3939.9*** | 5251*** | 1643.3*** |
|  | N rate (N) | 41.1*** | 70.7*** | 245.2*** | 268*** | 269.2*** |
|  | Variety (V) | 19.7*** | 5.2*** | 247.7*** | 211.7*** | 454.8*** |
|  | D×N | 0.7ns | 1ns | 1.6ns | 2.2ns | 5.7** |
|  | D×V | 60*** | 33.3*** | 356.4*** | 669*** | 230.7*** |
|  | N×V | 3* | 2.9ns | 14*** | 5.8** | 1.1ns |
|  | D×N×V | 3.2* | 9.9*** | 8.5*** | 4.3* | 7.4** |
| 2015 | ANOVA |  |  |  |  |  |
|  | Density (D) | 2464.8*** | 2892.8*** | 1953.3*** | 1301.5*** | 982.5*** |
|  | N rate (N) | 96.6*** | 143.9*** | 150.3*** | 197.3*** | 350.3*** |
|  | Variety (V) | 63.3*** | 27.2*** | 128.2*** | 165.5*** | 281.4*** |
|  | D×N | 6** | 11.3*** | 17.6*** | 26.1*** | 11.3*** |
|  | D×V | 11.6** | 3.1ns | 5.7 | 43*** | 41.7*** |
|  | N×V | 2.7ns | 3.7* | 4.3* | 2.2ns | 16.6*** |
|  | D×N×V | 3.1* | 5.9** | 1.2ns | 4.3* | 8.5*** |

Note:

^ns^ Not significance.

^*^ Significant at the 0.05 probability level.

^**^ Significant at the 0.01 probability level.

^***^ Significant at the 0.001 probability level.
