# Supplementary material for: Maize Canopy Apparent Photosynthesis and 13C-Photosynthate Reallocation in Response to Different Density and N Rate Combinations
Source: Front Plant Sci. 2019 Sep 19;10:1113. doi: 10.3389/fpls.2019.01113 (PMC6761910; doi:10.3389/fpls.2019.01113)
Supplement: Supplementary Table 4 — Analysis of variance of canopy apparent photosynthesis (CAP) at different growth stages as affected by density, N rate, and variety. [file Table_4.doc]

Supplementary Material

**Canopy apparent photosynthesis and ^13^C-photosynthate reallocation are vital to maize yield formation under different density and N rate combinations**

**Shanshan Wei****^1, 2^, Xiangyu Wang^2, 3^, Guanghao Li^2^, Dong Jiang^1, *^, Shuting Dong^2, *^**

***Correspondence:** Dong Jiang ([jiangd@njau.edu.cn](mailto:jiangd@njau.edu.cn))**;** Shuting Dong ([stdong@sdau.edu.cn](mailto:stdong@sdau.edu.cn))

**Supplementary Table 4** Analysis of variance of canopy apparent photosynthesis (CAP) at different growth stages as affected by density, N rate, and variety.

|  | CAP (μmol CO_2_ m^-2^ s^-1^) | | | | | | | |
| --- | --- | --- | --- | --- | --- | --- | --- | --- |
| Variation | 2013 | | | 2014 | | 2015 | | |
|  | VT | 20DAT | 40DAT | VT | 20DAT | VT | 20DAT | 40DAT |
| ANOVA |  |  |  |  |  |  |  |  |
| Density (D) | 128.1*** | 244.1*** | 67.9*** | 239.2*** | 220.8*** | 173.9*** | 157.7*** | 195.2*** |
| N rate (N) | 29.4*** | 80.4*** | 71.6*** | 219.7*** | 211.9*** | 133.0*** | 186.4*** | 151.6*** |
| Variety (V) | 0ns | 79.9*** | 210.8*** | 10.1** | 21.7** | 3.3ns | 67.7*** | 97.9*** |
| D×N | 1.9ns | 3.7* | 5.7** | 1ns | 5.8** | 8.1*** | 4.1* | 3.6* |
| D×V | 2.9ns | 0.4ns | 0.6ns | 0.2ns | 17.8*** | 4.7* | 1.7ns | 13.5** |
| N×V | 0.2ns | 1.6ns | 6.3** | 5** | 1.4ns | 1.9ns | 1.8ns | 2.8* |
| D×N×V | 0.7ns | 0.9ns | 0.5ns | 0.6ns | 1.6ns | 0.6ns | 0.6ns | 0.9ns |

Note: VT, 20DAT and 40DAT represent tasseling stage, 20 and 40 days after tasseling, respectively.

^ns^ Not significance.

^*^ Significant at the 0.05 probability level.

^**^ Significant at the 0.01 probability level.

^***^ Significant at the 0.001 probability level.
