# Supplementary material for: Maize Canopy Apparent Photosynthesis and 13C-Photosynthate Reallocation in Response to Different Density and N Rate Combinations
Source: Front Plant Sci. 2019 Sep 19;10:1113. doi: 10.3389/fpls.2019.01113 (PMC6761910; doi:10.3389/fpls.2019.01113)
Supplement: Supplementary Table 5 — Analysis of variance of RUBPCase and PEPCase activities at different growth stages as affected by density, N rate and variety. [file Table_5.doc]

Supplementary Material

**Canopy apparent photosynthesis and ^13^C-photosynthate reallocation are vital to maize yield formation under different density and N rate combinations**

**Shanshan Wei****^1, 2^, Xiangyu Wang^2, 3^, Guanghao Li^2^, Dong Jiang^1, *^, Shuting Dong^2, *^**

***Correspondence:** Dong Jiang ([jiangd@njau.edu.cn](mailto:jiangd@njau.edu.cn))**;** Shuting Dong ([stdong@sdau.edu.cn](mailto:stdong@sdau.edu.cn))

**Supplemental Table 5** Analysis of variance of RUBPCase and PEPCase activities at different growth stages as affected by density, N rate and variety.

| Factor | RUBPCase (μmol g^-1^ protein min^-1^) | | |  | PEPCase (μmol g^-1^ protein min^-1^) | | |  |
| --- | --- | --- | --- | --- | --- | --- | --- | --- |
|  | VT | | 20DAT | | VT | | 20DAT | |
|  | 2014 | 2015 | 2014 | 2015 | 2014 | 2015 | 2014 | 2015 |
| ANOVA |  |  |  |  |  |  |  |  |
| Density (D) | 531.3*** | 412.1*** | 444.9*** | 1074.2*** | 155.6*** | 299.1*** | 373.8*** | 324.8*** |
| N rate (N) | 197.7*** | 187.6*** | 252.8*** | 764.7*** | 46.3*** | 160.4*** | 87.2*** | 313.1*** |
| Variety (V) | 82.7*** | 18.4*** | 0.2ns | 1.1ns | 1ns | 46.2*** | 374.2*** | 255.1*** |
| D×N | 3.9* | 0.2ns | 7.1** | 1.4ns | 3.1* | 4.6** | 1.4ns | 3.5* |
| D×V | 5.5* | 11.1** | 46.8*** | 129*** | 0ns | 2.6ns | 1.2ns | 0.1ns |
| N×V | 3.3* | 4.6** | 3.4* | 11.1*** | 1.3ns | 4.4* | 5.6** | 1.9ns |
| D×N×V | 1ns | 0.9ns | 2.2ns | 1.3ns | 0.3ns | 0.8ns | 1.2ns | 1.0ns |

^ns^ Not significance.

^*^ Significant at the 0.05 probability level.

^**^ Significant at the 0.01 probability level.

^***^ Significant at the 0.001 probability level.
