# Supplementary figures and images for: Maize Canopy Apparent Photosynthesis and 13C-Photosynthate Reallocation in Response to Different Density and N Rate Combinations
Source: Front Plant Sci. 2019 Sep 19;10:1113. doi: 10.3389/fpls.2019.01113 (PMC6761910; doi:10.3389/fpls.2019.01113)

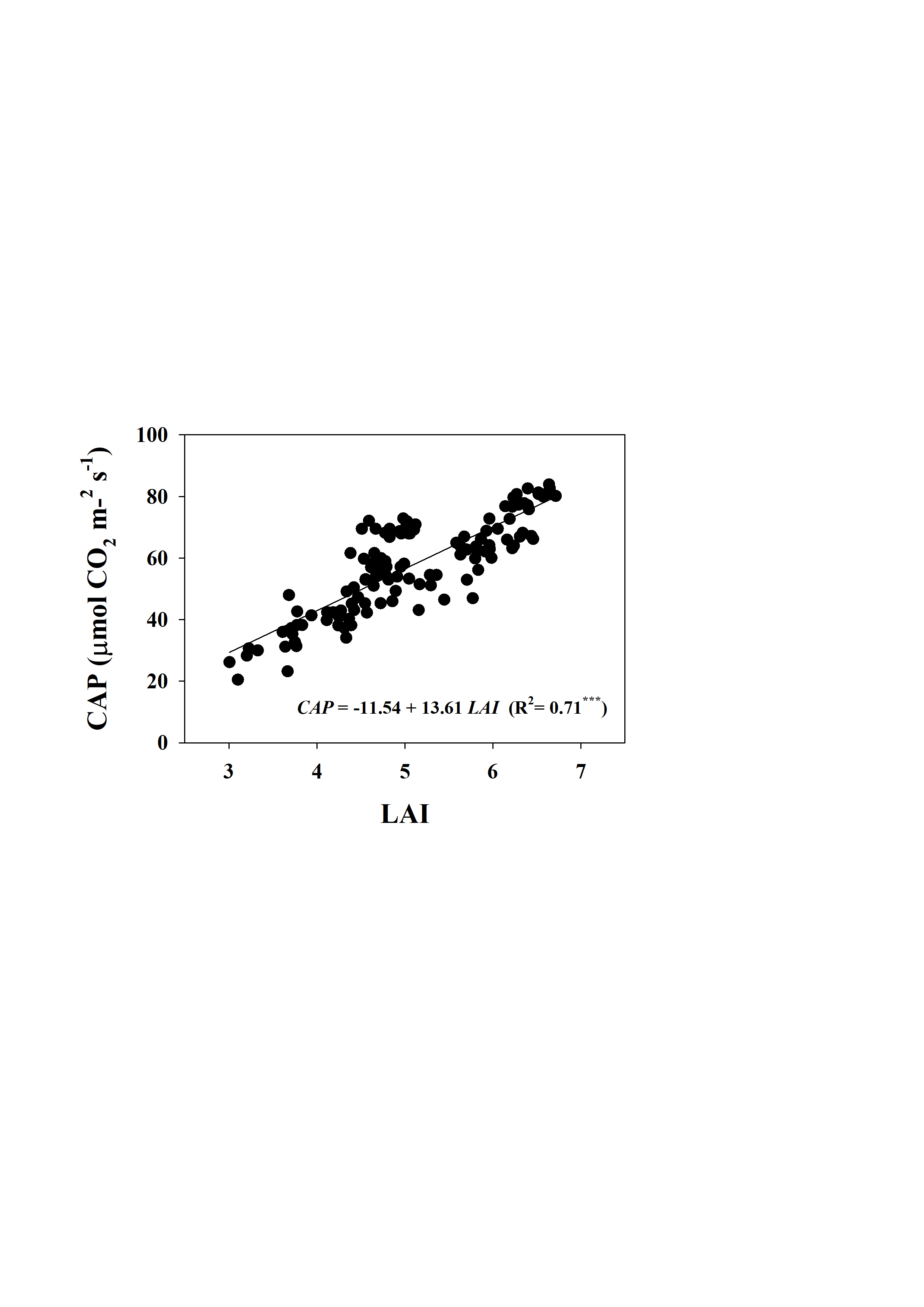

Supplement: Supplementary Figure S1 — Relationship between leaf area index (LAI) and canopy apparent photosynthesis (CAP). The data shown in figure were LAI and CAP values of the two varieties grown at low and high densities with different levels of nitrogen during 2013–2015 (n = 128). Asterisks (***) represent significance at the 0.001-probability level. [file Image_1.jpeg]

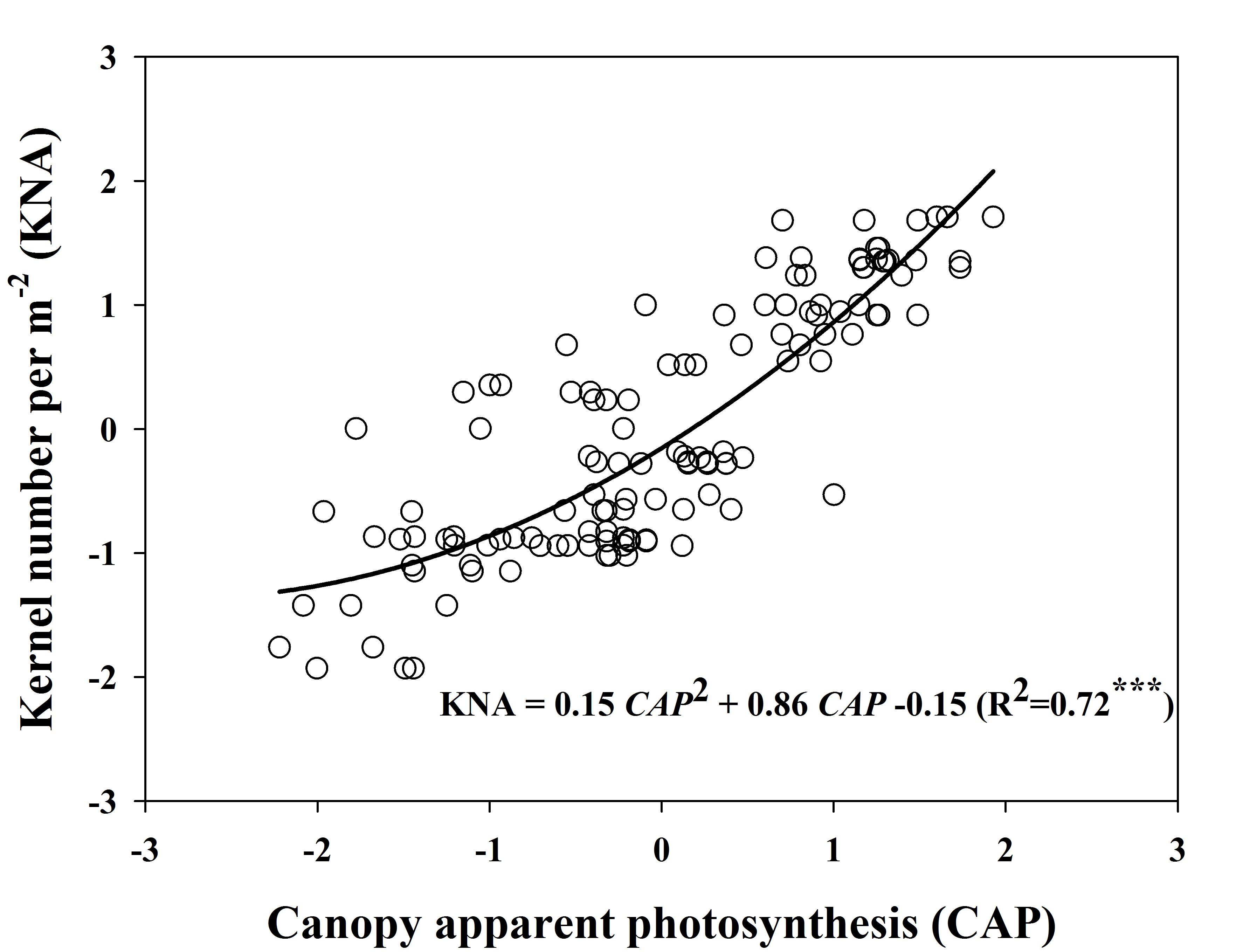

Supplement: Supplementary Figure S2 — Relationship between canopy apparent photosynthesis (CAP) and kernel number per unit area (KNA). The data shown in the figure was the standardized CAP and KNA values of the two varieties grown at low and high densities with different levels of nitrogen during 2013–2015 (n = 128). Asterisks (***) represent significance at the 0.001-probability level. [file Image_2.jpeg]

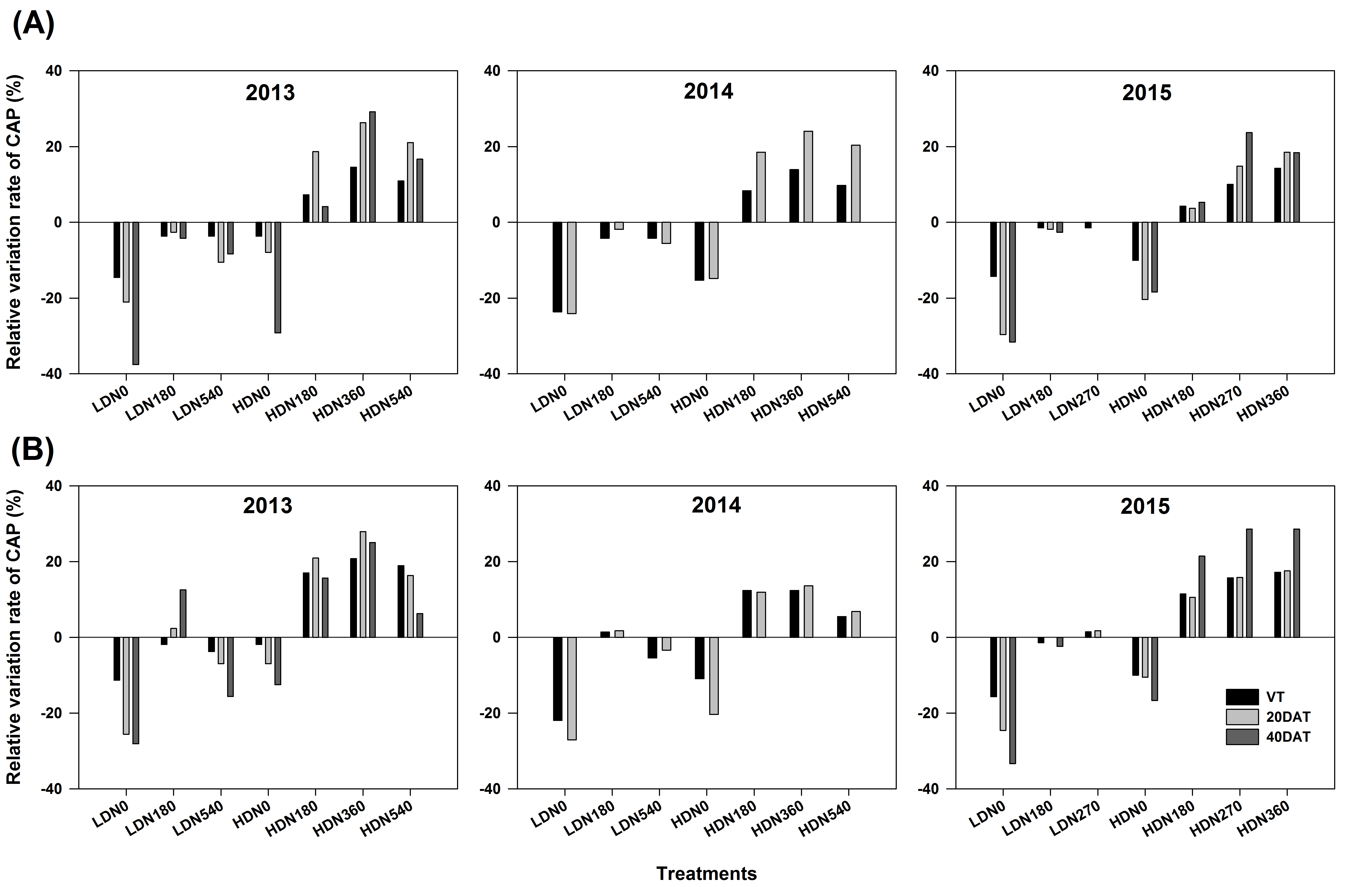

Supplement: Supplementary Figure S3 — The relative variation ratio of canopy apparent photosynthesis (CAP, %) under different density and nitrogen application rate combinations compared to LDN360 of DH618 (A) and DH605 (B) during the 2013–2015 growing seasons. LD and HD refer to low density and high density; N0, N180, N270, N360, and N540 represent nitrogen rates of 0, 180, 270, 360, and 540 kg ha–1, respectively VT, 20DAT, and 40DAT represent the tasseling stage, and 20 nd 40 days after tasseling, respectively. [file Image_3.jpeg]
